# Bruceine D Identified as a Drug Candidate against Breast Cancer by a Novel Drug Selection Pipeline and Cell Viability Assay

**DOI:** 10.3390/ph15020179

**Published:** 2022-01-31

**Authors:** Claudia Cipriani, Maria Pires Pacheco, Ali Kishk, Maryem Wachich, Daniel Abankwa, Elisabeth Schaffner-Reckinger, Thomas Sauter

**Affiliations:** 1 Systems Biology Group, Department of Life Sciences and Medicine, University of Luxembourg, L-4365 Esch-sur-Alzette, Luxembourg; claudia.cipriani1307@gmail.com (C.C.); maria.pacheco@uni.lu (M.P.P.); ali.kishk@uni.lu (A.K.); 2 Cancer Cell Biology and Drug Discovery Group, Department of Life Sciences and Medicine, University of Luxembourg, L-4365 Esch-sur-Alzette, Luxembourg; maryemwachich@gmail.com (M.W.); daniel.abankwa@uni.lu (D.A.); elisabeth.schaffner@uni.lu (E.S.-R.)

**Keywords:** natural products, bruceine D, emodin, scutellarein, drug prediction workflow, metabolic modelling

## Abstract

The multi-target effects of natural products allow us to fight complex diseases like cancer on multiple fronts. Unlike docking techniques, network-based approaches such as genome-scale metabolic modelling can capture multi-target effects. However, the incompleteness of natural product target information reduces the prediction accuracy of in silico gene knockout strategies. Here, we present a drug selection workflow based on context-specific genome-scale metabolic models, built from the expression data of cancer cells treated with natural products, to predict cell viability. The workflow comprises four steps: first, in silico single-drug and drug combination predictions; second, the assessment of the effects of natural products on cancer metabolism via the computation of a dissimilarity score between the treated and control models; third, the identification of natural products with similar effects to the approved drugs; and fourth, the identification of drugs with the predicted effects in pathways of interest, such as the androgen and estrogen pathway. Out of the initial 101 natural products, nine candidates were tested in a 2D cell viability assay. Bruceine D, emodin, and scutellarein showed a dose-dependent inhibition of MCF-7 and Hs 578T cell proliferation with IC_50_ values between 0.7 to 65 μM, depending on the drug and cell line. Bruceine D, extracted from *Brucea javanica* seeds, showed the highest potency.

## 1. Introduction

Breast cancer is one of the leading cancers in women, with around 2.26 million cases worldwide in 2020, representing an incidence of 11.7% and a mortality of 6.9% of all diagnosed cancers [1]. Breast cancer is a heterogeneous disease with various molecular features, clinical outcomes, and degrees of chemotherapy resistance. In order to provide a tailored treatment, breast carcinomas can be subdivided into five different molecular subtypes based on the presence of hormone receptors (HR, corresponding to estrogen receptor, ER, and progesterone receptor, PR), human epidermal growth factor receptor 2 (HER2), and the protein Ki-67, which serves as a marker for cellular proliferation: luminal A, normal-like (similar to luminal A, but with a slightly worse prognosis), luminal B, HER2-enriched, and triple-negative, which is also referred to as basal-like [2]. Typically, the luminal A subtype has a better prognosis, whereas the triple-negative subtype has the worst 5-year survival rate [3]. Breast cancer can further be clinically classified as a function of its size, location, and invasiveness. This classification comprises stage 0 (carcinoma in situ), and then ranges from stage I to stage IV, with the latter corresponding to invasive cancer that has propagated to distant sites of the body [4]. Besides the inter-tumour heterogeneity between patients, the clonal heterogeneity of a single breast tumour can cause therapies to fail to eradicate the whole tumour; hence, residual tumour cells tend to develop chemoresistance and metastasis [5].

The multi-target and heterogenous pharmacological mechanisms of action of natural products—and thus, their more ubiquitous efficiency—help to counteract cancer heterogeneity and prevent drug resistance [6]. Natural products, extracted from plants or animals, have been used to treat many diseases in various regional traditional medicines [7]. Nowadays, natural products including isolated herbal compounds and botanical mixtures containing a plethora of plant ingredients, as well as semi-synthetic derivatives and mimics, are being studied extensively for the treatment of different diseases, including cancer. To date, only two botanicals have been reviewed and approved by the FDA (sinecatechins and crofelemer) [8]. On the other hand, more single herbal compounds are used in clinical practice, such as paclitaxel and cabazitaxel for cancer treatment. These single herbal compounds are either extracted from plants or produced at an industrial scale in a semi-synthesis or total synthesis process, such as paclitaxel. Notably, some studies on breast cancer cell lines have shown synergistic effects following combined treatment with natural products and estrogen receptor modulators such as genistein and ormeloxifene [9], or equol and tamoxifen [10].

In silico metabolic models allow the simulation of the metabolism of a whole cell or an organism [11], both for different metabolic phenotypes [12] and diseases, such as cancer [13,14,15]. Metabolic models were recently used to speed up the process of identifying possible new applications for existing drugs [15,16]. In the present study, we present a new drug prediction and analysis pipeline (https://github.com/sysbiolux/Herbal_drug_prediction accessed date 14 December 2021) tailored to the complex mode of action of natural products and the lack of the complete knowledge of their targets. The pipeline requires the gene expression data of cells treated with natural products and a genome-scale reconstruction such as Recon3D as an input in order to build context-specific metabolic models via FASTCORMICS or rFASTCORMICS [15,17]. The models are then used to simulate the effects of the natural products on the metabolism of cancer cells, and to prioritise the natural products based on their predicted efficacy. Here, microarray gene expression data of the MCF-7 breast cancer cell line treated with 101 natural products [18] were used as the input for the pipeline. Out of 101 single natural products, and one mixture of natural products, nine products were identified as promising candidates with anticancer activity, and were subjected to experimental validation via a 2D cell viability test, which is commonly used in the drug discovery process. Among the nine candidates, emodin, scutellarein, and bruceine D caused a decrease in the proliferation of MCF-7 and Hs 578T cells at low IC_50_ values, indicating the good performance of the developed drug prediction pipeline.

## 2. Results

In order to assess the potential effect of natural products on the metabolism of breast cancer cells, and to identify promising drug candidates, a microarray expression dataset composed of the expression data of MCF-7 cells treated with 101 natural products, one mixture of natural products (salvianic acid A sodium, salvianolic acid B, protocatechuic aldehyde and tanshinone IIA), and the control—dimethyl sulfoxide (DMSO) [18]—was used to reconstruct context-specific metabolic models (102 natural product models and a control model) via the FASTCORMICS workflow [17]. The median, minimal and maximal number of reactions, metabolites, and genes of the models are displayed in Table 1. The size of the models varies between 1593 and 2169 reactions, with a median of 1895 reactions. An in silico drug prediction and analysis workflow composed of (i) the drug prediction of growth by natural products’ target deletion, (ii) the assessment of the dissimilarity of the included reactions compared to the DMSO (control) model reactions, (iii) the assessment of the similarity of the predicted flux ranges carried by the reactions of natural product models and the DMSO model constrained by the approved breast cancer drugs, and (iv) a pathway analysis, which was used on these 103 models to predict 23 potential candidates. The model statistics for all of the 102 conditions (101 natural products + one mixture) are available in Table S1 of Supplementary File S1. However, in order to facilitate the representation of the data, only the data of the predicted 23 drugs are shown in the main text.

### 2.1. Ferulic Acid, Resveratrol, Capecitabine, and Methotrexate Are Predicted to Reduce the Growth of MCF-7 Cells

Among these 101 natural products, human targets for only 44 drugs were found in DrugBank V5 [19], PROMISCUOUS 2.0 [20], and NPASS [21]; 35 drugs thereof targeted metabolic genes (see Supplementary File S1, Table S2 for the detailed drug–target interactions, and Supplementary File S2, Table S3 for the summary of the number of interactions). The effect of these 35 natural products on the growth of MCF-7 cells was simulated in silico by setting the bounds of all of the target reactions in the control DMSO model to zero. After the knockout of the targets of ferulic acid and resveratrol, the DMSO model could not produce biomass anymore. As a quality control, 26 approved breast cancer drugs (see Supplementary File S1, Table S4 for the list of breast cancer drugs, and Supplementary File S1, Table S5 for the detailed drug–target interactions) were tested using the same approach on the DMSO model. Capecitabine, a precursor of the drug 5-fluorouracil (5-FU) [22], and methotrexate, an antagonist of folic acid [23], were predicted to stop the growth of cancer cells. The other 24 tested breast cancer drugs had a predicted growth ratio of more than 0.998, suggesting that the other 24 breast cancer drugs have modes of action that do not target the metabolism of cancer cells, or target pathways that are not directly related to growth. In order to assess whether the other 24 breast cancer drugs could have a combined effect with natural products, two different experiments were performed. First, the targets of every combination of natural product and breast cancer drug in the DMSO model were set to zero, in order to assess whether the combination of the two drugs caused a stronger reduction of the growth than each natural product or cancer drug individually. Second, in order to verify that the natural products do not have undescribed targets that might result in synergies when used in combination with breast cancer drugs, the targets of the breast cancer drugs were set to zero in the reconstructed 102 natural product models (101 single drugs and one mixture of four drugs). However, there was no significant reduction of the growth when we compared the cancer drug deletion on the DMSO model and the natural product model. The single-gene deletion was applied to ferulic acid, resveratrol, methotrexate, and capecitabine in order to determine which of these drug targets can decrease the biomass individually. Single-gene deletion did not predict any single essential genes for ferulic acid or resveratrol but identified three single essential genes for methotrexate (Thymidylate synthase (TYMS), Dihydrofolate reductase (DHFR), 5-Aminoimidazole-4-Carboxamide Ribonucleotide Formyltransferase (ATIC)) and one single essential gene (TYMS) for capecitabine.

### 2.2. Bruceine D, Narciclasine, Hydroxysafflor Yellow A, Ferulic Acid, and Salvianolic Acid B Are Predicted to Show the Strongest Perturbations of the Metabolism of MCF-7 Cells

**Figure 1:**
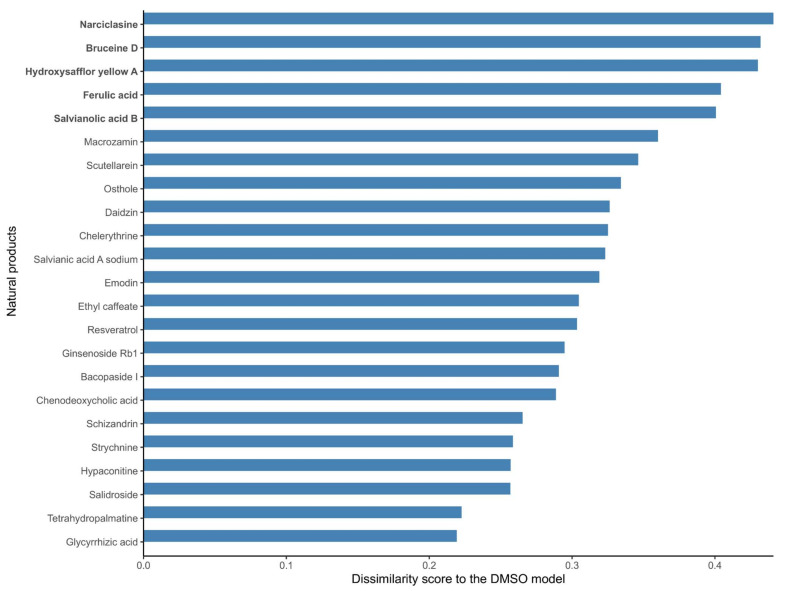
Dissimilarity scores between the breast cancer models reconstructed from the expression data of MCF-7 cells treated with natural products and the DMSO model (control model). Natural products that alter the metabolism of breast cancer cells to a large extent (in bold) have a high dissimilarity to the control DMSO model. A dissimilarity score (D) was computed based on the Jaccard Similarity index (J, where J is the number of shared reactions between two models divided by the total number of reactions), where D is equal to 1-J. The dissimilarity score represents the fraction of reactions that are different between the two models.

As mentioned before, no metabolic target information could be found in DrugBank V5 [19], PROMISCUOUS 2.0 [20], or NPASS [21] for 66 out of the 101 natural products, and the list of targets of the 35 listed drugs is likely to be incomplete, as these drugs have multiple targets. Hence, in order to further identify candidate natural products that might affect the metabolism of MCF-7 cells, the structure of the reconstructed natural product models in terms of the included reactions in the natural product models was compared to the DMSO control model. A dissimilarity score was computed for each natural product model against the DMSO model (using 1- Jaccard Index J, where J is the number of shared reactions between a natural product and the DMSO model divided by the total number of reactions). A dissimilarity score of 1 means that the natural product model does not share any reaction with the DMSO model; hence, the associated drug has a strong effect on the metabolism of MCF-7 cells. On the other hand, a score of 0 means that the two models are identical; hence, the effect of the natural product drug on the MCF-7 metabolism is negligible in terms of its network structure. The calculated dissimilarity score ranged from 0.19 to 0.44 for ephedrine hydrochloride and narciclasine, respectively. The group of the top five natural product models (narciclasine, bruceine D, hydroxysafflor yellow A, ferulic acid, and salvianolic acid B) with the highest dissimilarity scores in the DMSO model (ranging from 0.44 to 0.40) were retained for further analysis. Figure 1 shows the dissimilarity values for the 23 drugs predicted as potential candidates by at least one of the steps of the drug prediction workflow (Table 2), and Supplementary Figure S1 contains the complete results. The fifth and sixth top-ranked drugs, salvianolic acid B and nitidine chloride, have dissimilarity scores of 0.40 and 0.37, respectively, which are larger differences compared to the drugs higher ranks (Supplementary Figure S1).

### 2.3. 13 Natural Products Are Predicted to Affect the Metabolism of MCF-7 Cells Similarly to the Breast Cancer Drugs Capecitabine and Methotrexate

With a high dissimilarity score for a natural product model compared to the DMSO model does not necessarily imply that the associated natural product is efficient against breast cancer, but rather that the metabolic changes induced by the natural products are different from the ones induced by DMSO. However, selecting natural products that have a similar effect to known breast cancer drugs on the metabolic models increases the likelihood of the identification of efficient drug candidates.

**Figure 2:**
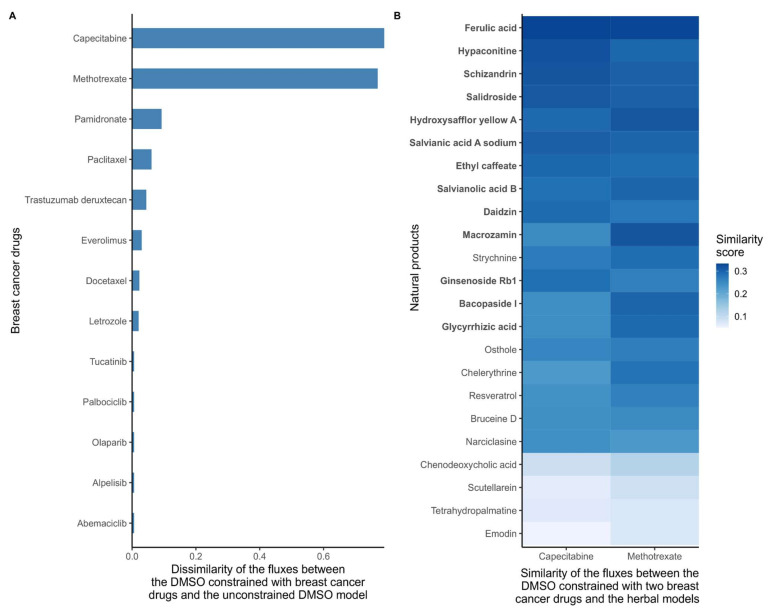
(**A**) Dissimilarity of the flux ranges between the DMSO models constrained with breast cancer drugs and the unconstrained DMSO model. Breast cancer drugs that significantly alter the metabolism of MCF-7 cells have a high dissimilarity to the control DMSO model. With low dissimilarity scores suggests that the main targets of some drugs are non-metabolic, that these drugs have indirect effects on growth, or that the drugs cause cancer death by affecting pathways that are unrelated to growth. The dissimilarity score was computed based on the flux ranges of the reactions included in the models. If both models maintain similar ranges, the dissimilarity score is close to 0. (**B**) Similarity scores between the reconstructed natural product models and the DMSO model constrained with capecitabine and methotrexate. The target deletion of these two drugs is predicted to stop the growth of MCF-7 cells. Hence, natural product models with a high similarity with DMSO constrained with these drugs are more likely to have a similar effect on the metabolism. The similarity score was computed based on the flux ranges of the reactions included in the models. If both models maintain similar ranges, then the similarity score tends to 1. The natural products were sorted by the average similarity score to methotrexate and capecitabine, with the ten natural products with the highest similarity to capecitabine and the ten drugs with the highest similarity score to methotrexate are highlighted in bold.

As a first step, and in order to quantify the effect of all 26 breast cancer drugs, the dissimilarity score between the DMSO model constrained by the cancer drug targets (cancer drug models) and the unconstrained DMSO model was computed based on flux ranges obtained using Flux Variability Analysis (FVA). Models that included reactions that maintained the same flux range between two models have a dissimilarity score of 0, whereas models that have very different flux ranges will receive a score close to 1. Methotrexate and capecitabine had the highest dissimilarity scores with the DMSO model (Figure 2A), which is consistent with the strong effect of these drugs in the growth prediction step above. Then, the similarity score (1, dissimilarity score) between the natural product models and two DMSO models constrained by methotrexate and capecitabine (cancer drug models) were calculated. The combined top-ten natural product models which were similar to methotrexate and capecitabine (resulting in 13 unique natural products) were selected as candidate anticancer drugs. The results for these 13 natural product models are shown in (Figure 2B).

### 2.4. Narciclasine, Emodin, Scutellarein, Strychnine, Resveratrol, Chenodeoxycholic Acid, Chelerythrine, Tetrahydropalmatine, Osthole, and Glycyrrhizic Acid Are Predicted to Alter the Androgen and Estrogen Synthesis and Metabolism Pathway

**Figure 3:**
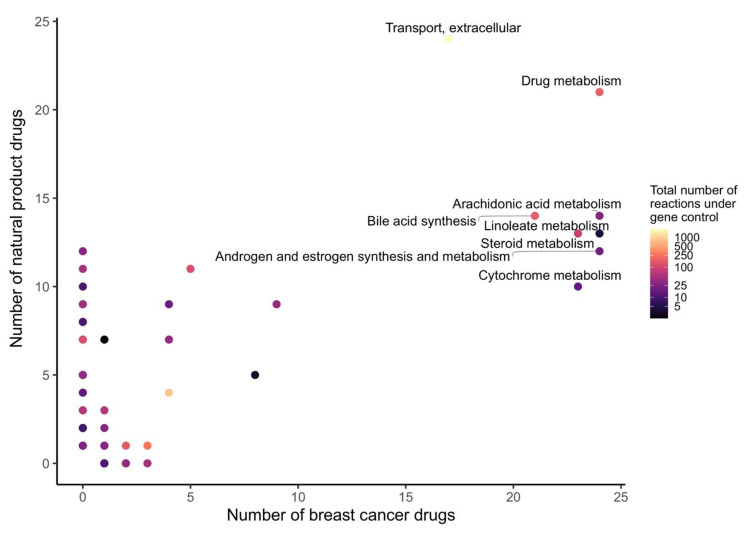
Androgen and estrogen synthesis and metabolism, arachidonic acid metabolism, cytochrome metabolism, drug metabolism, bile acid synthesis, steroid metabolism, linoleate metabolism, and extracellular transport are the most frequently targeted pathways of approved breast cancer drugs and natural products. The x- and y-axes correspond to the number of breast cancer drugs and natural products that target Recon3D. The colour map represents the total number of reactions in each pathway under gene control.

**Figure 4:**
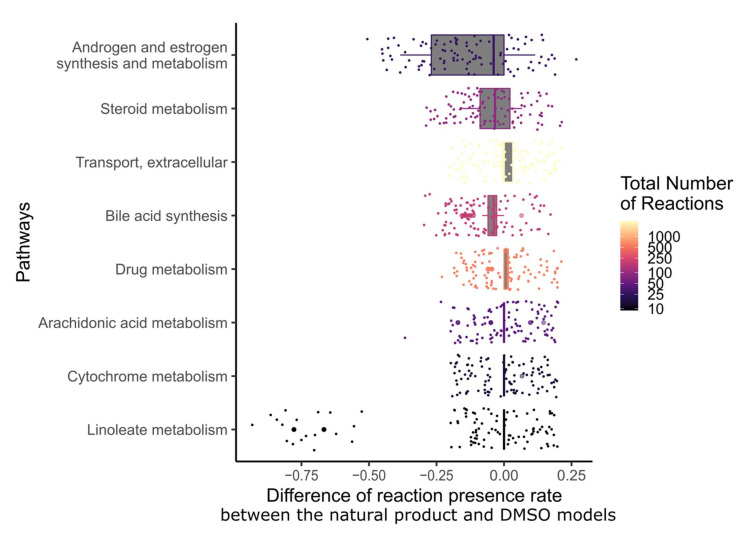
Among the pathways that are targeted by most breast cancer drugs, androgen and estrogen synthesis and metabolism had the lowest fraction of shared reactions between the natural product and control models. The fraction of reactions present per pathway for the eight most frequently targeted pathways of breast cancer drugs was calculated for each natural product by dividing the number of present reactions in each context model by the total number of reactions in this pathway. Then, the difference in reactions present per pathway between the natural product models and the DMSO model was computed. The pathways were sorted by the interquartile range of the differences in the fraction of present reactions. The colour code corresponds to the total number of reactions in each pathway.

In order to determine which metabolic pathway should be preferably targeted in order to reduce the growth of breast cancer, and which pathways are targeted by natural products, the drug targets of natural products and breast cancer drugs were mapped to the reaction–gene matrix (rxnGeneMat field) of Recon3D [24], the input reconstruction which was used to build the natural product models. The number of breast cancer drugs targeting the same pathway was plotted against the number of natural products (Figure 3). Most breast cancer and natural products target eight pathways: androgen and estrogen synthesis and metabolism, arachidonic acid metabolism, drug metabolism, bile acid synthesis, steroid metabolism, cytochrome C metabolism, linoleate metabolism, and extracellular transport pathways. The colour code on the plots represents the size of the pathway (the number of reactions under gene control in this pathway, which can therefore be targeted). Larger pathways such as extracellular transport and drug metabolism, which have 332 and 55 associated genes, respectively, are expected to be targeted by a higher number of drugs than smaller pathways. In order to verify whether the metabolism of these pathways was significantly altered by the natural products, the difference in the active reactions (the reactions present in the models) between each natural product model and the DMSO model was computed. Among the eight pathways that are targeted by most approved breast cancer drugs (Figure 3 and Figure 4), the androgen and estrogen synthesis and metabolism pathway had the lowest rate of reactions that were shared between the natural product models and the DMSO model, i.e., reactions that were active in the DMSO model tended to be inactive in the natural product models, and vice versa. Hence, this suggests that natural product models more strongly impact this pathway. The same analysis was performed on all of the pathways in Recon3D. Among the pathways with the highest rate of altered reactions are pathways with a low overall number of reactions. For pathways, with less than or equal to five reactions, the activation or inactivation of a single reaction will result in a rate of altered reactions that is above or equal to 0.2, whereas for larger pathways the alteration of one reaction would have a more modest effect (see the colour code for the size of the pathways). The choice of the androgen and estrogen synthesis and metabolism pathway as the pathway of interest for breast cancer treatment is consistent with three facts. First, the inhibition of Estrogen Receptor α (ERα) by fulvestrant was shown to prevent invasion by MCF-7 cells, which are ER-positive, in a xenograft experiment [25]. Second, genistein, which also targets ERα, inhibits the proliferation of MCF-7 cells and induces apoptosis [26]. Third, estrogen- and androgen-based therapeutics are used for breast cancer treatment [27].

In total, 12 natural products were described to have targets in the androgen and estrogen synthesis and metabolism pathway. However, only ten out of these twelve showed a difference in the fraction of reactions present per pathway when compared to the DMSO model (see Supplementary File S2, Figure S4): narciclasine, emodin, scutellarein, strychnine, resveratrol, chenodeoxycholic acid, chelerythrine, tetrahydropalmatine, osthole, and glycyrrhizic acid.

### 2.5. 23 Natural Products with Potential Anticancer Activity Emerged from the Different Stages of Analysis

In total, 23 out of the 101 natural products and one drug mixture were predicted as the top candidates in at least one of the four in silico analysis steps: (i) the drug deletion prediction by the natural products’ targets, (ii) the dissimilarity to the DMSO model, (iii) the similarity to breast cancer drugs, and (iv) pathway analysis. This number was further reduced for the experimental validation by selecting only those natural products that popped up in more than one of the in silico steps (Table 2, green). Notably, strychnine and narciclasine were removed from the list due to toxicity (Table 2, red) [28], and due to the abundance of available experimental data (Table 2, orange, see Supplementary File S1, Table S6) on breast cancer cell lines, respectively [29]. Additionally, emodin, salvianic acid A sodium, scutellarein, and bruceine D (Table 2, blue)—which were predicted by only one analysis step—were added to the list of drugs for validation due to previous evidence from two databases (Dr. Duke’s Phytochemical and Ethnobotanical Databases (Dr. Duke Database) and the Drug Repurposing Hub Database) [30,31] and from the literature [32]. This resulted in a final list of nine drugs for the experimental validation.

### 2.6. Scutellarein, Emodin, and Bruceine D Decrease the 2D Cell Viability of MCF-7 and Hs 578T Breast Cancer Cells

In order to experimentally validate the in silico predictions, 2D cell viability assays were performed to determine the anticancer activity of the nine selected natural product candidates: scutellarein, emodin, bruceine D, resveratrol, hydroxysafflor yellow A, salvianolic acid B, salvianic acid A sodium, ferulic acid, and glycyrrhizic acid. Ophiobolin A, which is a natural product that covalently inhibits calmodulin and has anticancer stemness properties [33], was chosen as a control compound because of its anti-proliferative effects in different cancer cell lines [34]. Two breast cancer cell lines were used for these assays: the non-invasive MCF-7 cells, which are ER+ breast cancer cells that were also used for the computational analysis, and the highly invasive Hs 578T cells, which are triple-negative breast cancer cells.

**Figure 5:**
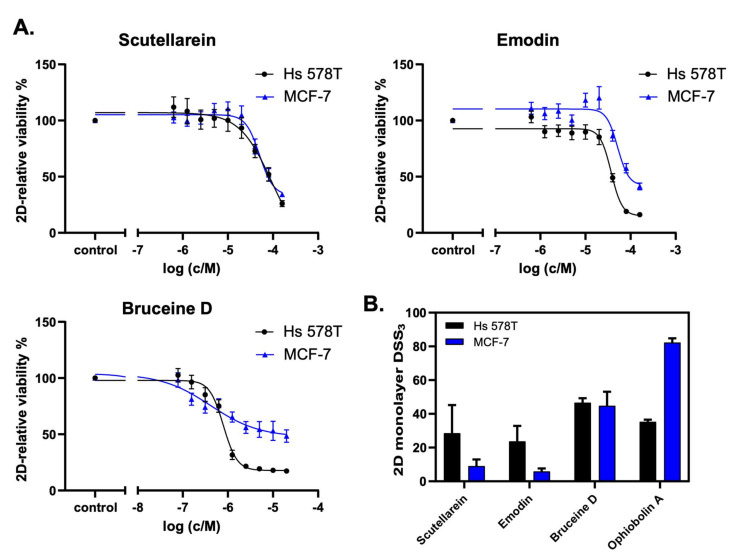
Scutellarein, emodin, and bruceine D reduce 2D breast cancer cell viability. (**A**) The natural products scutellarein, emodin, and bruceine D dose-dependently decrease the 2D cell viability of both MCF-7 and Hs 578T breast cancer cell lines after 72 h. The treatment was performed in a 1:2 dilution series ranging from 160 μM to 0.625 μM for scutellarein (*n* = 4) and emodin (*n* = 4), and ranging from 20 μM to 0.078 μM for bruceine D (*n* = 3). (**B)** The 2D monolayer DSS_3_ values as a quantification of the anti-proliferative effects of the natural products scutellarein, emodin, and bruceine D, and of the control compound, ophiobolin A. The DSS_3_ values correspond to the normalized area-under-the-curve values of the dose–response data, as shown under (**A**). The results are expressed as the mean +/- SEM, *n* ≥ 3 for natural products, and *n* = 2 for ophiobolin A.

The analysis of the cell viability revealed that scutellarein, emodin, and bruceine D inhibited the cell proliferation of both MCF-7 and Hs 578T cells in a concentration-dependent manner (Figure 5A). Notably, lower drug concentrations were used for bruceine D, as this compound showed the highest potency. Figure 5B shows the DSS_3_ values as determined using the DSS pipeline website, BREEZE (https://breeze.fimm.fi/ last accessed on 14 December 2021). DSS_3_ values essentially correspond to the normalized area under the curve of dose–response curves [35], with a higher DSS3 value reflecting a higher potency of the drug. The obtained DSS_3_ values point to a higher potency for bruceine D than for the control compound ophiobolin A in invasive Hs 578T cells, and to a similar potency of bruceine D in MCF-7 cells compared to Hs 578T cells (Figure 5B and Supplementary File S2, Figure S5A). Notably, bruceine D showed submicromolar IC_50_ (IC_50_ = 0.71 ± 0.05 μM) in Hs 578T, and low micromolar IC_50_ in MCF-7 cells (IC_50_ = 9.5 ± 7.7 μM) (Supplementary File S2, Table S9), which is in the same range. The other two candidates—scutellarein and emodin—gave IC_50_ values ranging from 28 to 65 μM depending on the compound and the cell line (Supplementary File S2, Table S9). The IC_50_ measured for the three natural products is in the same range for MCF-7 as the one found in the literature (Supplementary File S1, Table S10). No data were found for these three natural products for Hs 578T. On the other hand, treatment with hydroxysafflor yellow A, salvianolic acid B, salvianic acid A sodium, ferulic acid, or glycyrrhizic acid did not confirm the in silico predictions (Supplementary File S2, Figure S5B), as these natural products did not induce a dose-dependent decrease of 2D cell viability. Only resveratrol showed a minor dose-dependent effect on Hs 578T cells at high concentrations. Consistently, these six drugs gave very low DSS_3_ values, and no IC_50_ values could be determined (Supplementary File S2, Table S9). Altogether, our experimental validation indicates that the three natural products emodin, scutellarein, and bruceine D are promising therapeutic candidates for breast cancer treatment, as predicted by the computational model, with bruceine D being the top candidate.

### 2.7. Targeting the Androgen and Estrogen Synthesis and Metabolism, as well as the Accumulation of ROS, Are the Two Main Modes of Action of Emodin, Bruceine D and Scutellarein

**Figure 6:**
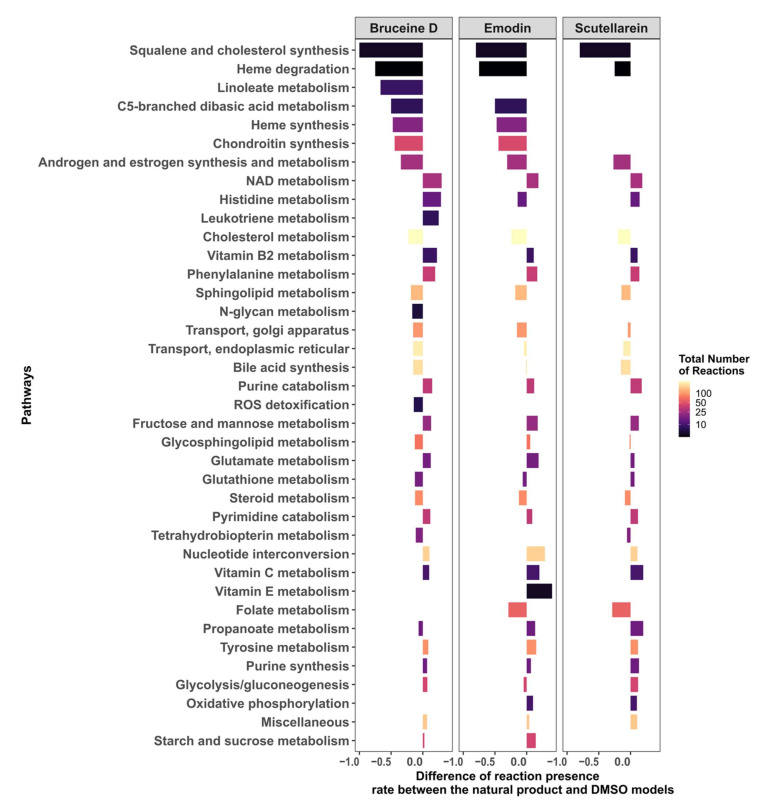
Androgen and estrogen synthesis and metabolism, and pathways implicated in the maintenance of redox homeostasis, are the main targets of bruceine D, emodin, and scutellarein.

In order to understand how emodin, bruceine D, and scutellarein affect the metabolism of MCF-7 cells, the difference in the reaction presence rates between the three respective natural product models and the DMSO model was calculated. The androgen and estrogen synthesis and metabolism pathway, as well as the pathways required for the biosynthesis of estrogens such as cholesterol, steroid and squalene, and cholesterol synthesis, had a lower presence rate in the three natural product models, suggesting that estrogen synthesis is downregulated by these three drugs (Figure 6; the results for all of the pathways are shown in Supplementary File S2, Figure S6). Furthermore, pathways directly implicated in redox homeostasis—such as Reactive Oxygen Species (ROS) detoxification (bruceine D), Heme degradation, and NAD metabolism—and pathways such as purine and pyrimidine, which are required to replenish the stocks of NAD(P)H, folate metabolism (emodin, scutellarein), and glutathione metabolism (bruceine D and emodin) were also downregulated. Transporters that indirectly reduce stress by transporting compounds that might cause redox stress out of the Golgi and endoplasmic reticulum are also targeted by the three drugs. Vitamins, phenylalanine, and histidine metabolism—which are pathways known to alleviate redox oxidative stress—were activated, suggesting that these pathways are activated in order to reduce the accumulation of ROS in MCF-7 cells.

The differences in the reactions present per pathway between the natural product and the DMSO models were calculated. Only pathways that have at least an absolute difference of 0.1 in the fraction of present reactions for bruceine D, scutellarein and emodin are displayed. The pathways are ranked in the *y*-axis by their absolute differences in reactions present per pathway for bruceine D. Supplementary File S2, Figure S6 shows the results for all of the pathways in Recon3D.

**Figure 7:**
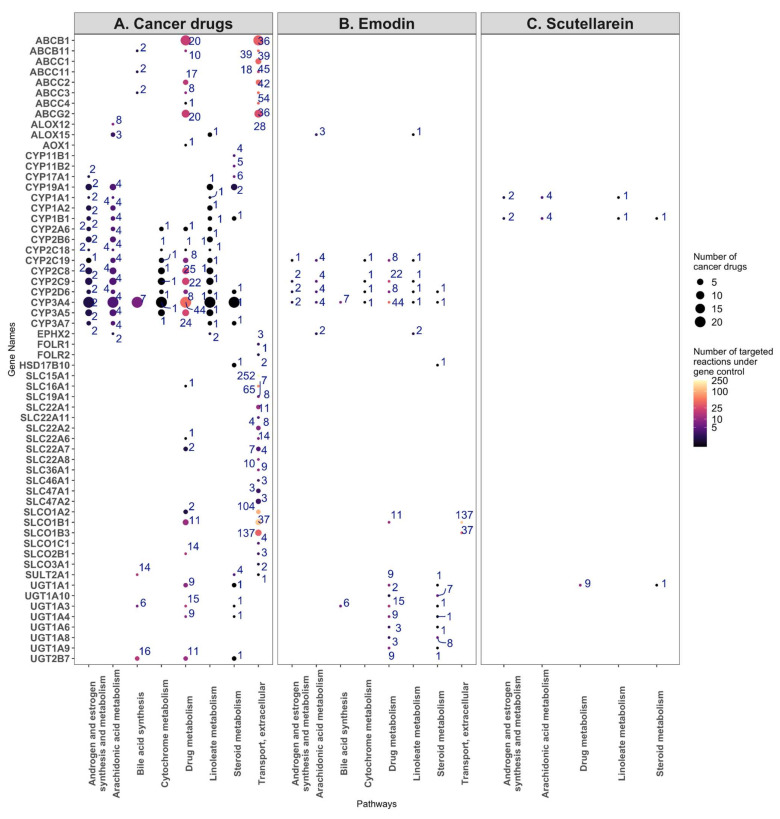
Target genes and the number of reactions affected by the breast cancer drugs (**A**), emodin (**B**), and scutellarein (**C**) for the eight most commonly targeted pathways. (**A**) The target genes of the breast cancer drugs were plotted against the eight most commonly targeted pathways, with the size of the dots indicating the number of cancer drugs targeting a gene, and the number near each dot and colour map indicating the number of reactions under the control of the gene. (**B**) The same analysis, but for the targets of emodin. (**C**) The same analysis, but for the targets of scutellarein.

In order to ascertain which enzymes are most likely targeted by bruceine D, emodin, and scutellarein, the number of reactions impacted in each pathway was plotted against the known drug targets for each compound. The size of the dots indicates how many cancer drugs target the same gene. Bruceine D has no described targets in DrugBank V5 [19] (version 5.1.8), PROMISCUOUS 2.0 [20], or NPASS [21]. Emodin has 24 metabolic targets—notably CYP3A4, CYP2C9, CYP2C19, CYP2D6—targeting, among others, the androgen and estrogen synthesis and metabolism pathway. These four genes are also targeted by 21, seven, three and two breast cancer drugs, respectively (Figure 7A). Scutellarein has nine metabolic targets, notably CYP1A1 and CYP1B1 in the androgen and estrogen synthesis and metabolism pathway, which are targeted by toremifene, and by paclitaxel and docetaxel, respectively (Figure 7B). Furthermore, 17 genes of the androgen and estrogen synthesis and metabolism pathway are targeted by breast cancer drugs. Notably, CYP3A4 is targeted by 21 drugs. The same gene controls reactions in the steroid, linoleate, drug, cytochrome C, bile acid, and arachidonic acid metabolism (Figure 7C). The 17 target genes in the androgen and estrogen synthesis and metabolism pathway (including CYP3A4) regulate four reactions that hydrolyse oestrone, testosterone, and pregnenolone into products that have been described to bind estrogen or androgen receptors and induce proliferation in breast cancer cells [36] (Supplementary File S1, Table S11, Supplementary File S2, Figure S8). In the arachidonic pathway and other pathways with the reactions controlled by the CYP proteins targeting breast cancer drugs, none or few reactions were differentially present in the natural product models, suggesting that these pathways were not targeted by natural product models (Supplementary File S2, Figure S8).

Besides the androgen and estrogen synthesis and metabolism pathway, emodin targets 12, 10, nine, six, and five genes in the drug, steroid, vitamin A, linoleic, and arachidonic metabolisms, respectively, whereas scutellarein targets three genes in the starch and sucrose metabolisms (Supplementary File S2, Figure S7).

The 26 cancer drugs mainly target CYP proteins, SLC carriers, ATP transporters, and UDP-glucuronosyltransferases, which are mainly found in the eight following pathways: androgen and estrogen synthesis and metabolism, arachidonic acid metabolism, bile acid synthesis, cytochrome metabolism, drug metabolism, linoleate metabolism, steroid metabolism, transport, and extracellular pathways. Some of these genes and pathways are also targeted by emodin. On the other hand, although scutellarein targets two CYP proteins and one UDP-glucuronosyltransferase, it does not target CYP3A1. Unlike emodin, which shares several targets with the breast cancer drugs, scutellarein has only a few targets in these pathways, suggesting a different model of action for this drug (Figure 6, Supplementary File S2, Figure S6).

## 3. Discussion

In the present work, a drug discovery workflow based on FASTCORMICS, Drug deletion, and Flux Variability Analysis was used to predict the best breast cancer drug candidates among 101 natural products, out of which nine were tested in a 2D cell viability assay using MCF-7 and Hs 578T cells. A dose-dependent decrease of cell viability was observed with the three natural products emodin, scutellarein, and bruceine D, with the latter showing a low micromolar IC_50_ in MCF-7 cells (IC_50_ = 9.5 ± 7.7 μM), and a submicromolar IC_50_ in Hs 578T cells (IC_50_ = 0.71 ± 0.05 μM).

Metabolic modelling was previously applied, with success, to the prediction of drug candidates and, more specifically, repurposable drugs [15,16]. However, the information for natural products is scarcer than that for conventional breast cancer drugs. Only 35 of the 101 drugs had target information in DrugBank V5, PROMISCUOUS 2.0, and NPASS (see Supplementary File S2, Table S3). Furthermore, due to the multi-target mode of action of natural products, the list of targets for the natural products present in these databases is probably incomplete. Hence, drug deletion approaches might miss interesting drug candidates as some of their targets might be missing from databases. Finally, some of these drugs that might not have direct metabolic targets could nevertheless have a significant effect on metabolism and cancer cell viability, growth, and migration.

In order to circumvent this issue, we identified natural products that, according to their expression profiles, affect the metabolism of cancer cells differently than the solvent DMSO alone. This approach does not necessarily imply that these products reduce growth, but at least it allows us to eliminate natural products with no or very small effects on the metabolism. We further selected products that have a similar effect to capecitabine and methotrexate on the metabolism of MCF-7 cells. These two breast cancer drugs had the strongest dissimilarity to the flux distribution of DMSO due to the shutdown of all of the reactions that are required for biomass production, suggesting that they have a strong impact on the metabolism. This assumption was further validated by the drug deletion that predicted these two drugs to reduce growth. Methotrexate has three metabolic targets that are predicted to reduce growth upon knockout: TYMS, DHFR, and ATIC. The knockout of TYMS was also predicted to stop growth in the capecitabine-constrained DMSO model. Because it is a rate-limiting enzyme of nucleotide synthesis, the knockdown of TYMS was previously described to reduce the proliferation of breast cancer cells [37]. The inhibition of TYMS by fluoropyrimidine and folate analogues was further shown to induce apoptosis through ROS generation [38]. Methotrexate and other DHFR inhibitors deplete the concentration of folates required for the de novo synthesis of nucleotides and NADP(P)H that regulates oxidative stress by the regeneration of reduced glutathione. Finally, ATIC is an oncogene that suppresses AMPK activation, and hence promotes proliferation via mTOR [39].

The knockout of the metabolic targets of the 24 remaining breast cancer drugs did not affect the growth in the MCF-7 models. Although the cancer drugs had around 82 combined metabolic targets, the targets responsible for the reduction in proliferation or cell death might be non-metabolic. These drug targets might, moreover, have an indirect effect on growth; hence, they would not be captured by constraining the DMSO models by the known targets. Furthermore, the metabolic impact might not be captured by the biomass formulation, i.e., by causing the accumulation of ROS and the death of the cell by apoptosis. Finally, all of the isozymes described to control a reaction in Recon3D might not be expressed in breast cancer cell lines, or were missing in the target list. Regarding the targets, ABCB1 and ABCG2—which are ATP-dependent drug efflux pumps for the xenobiotic compound, and are known to be linked to multidrug resistance [40]—were targeted by 17 and nine drugs, respectively. More generally, among the 82 combined targets, eight were ABC transporters, 13 were solute carriers, 16 were members of the CYP proteins, and six were members of the SLCO family of organic anion-transporting polypeptides. In Recon3, CYP34A—which is targeted by 21 breast cancer drugs—controls sex hormones and metabolizes 50% of cancer drugs. Hence, the overexpression of this CYP protein in cancer tissue might also be linked to multidrug resistance.

Among the natural product models, resveratrol and ferulic acid were predicted to decrease proliferation. However, unlike for methotrexate and capecitabine, the predicted growth reduction is not linked to the knockout of one gene, but to the combined effect of the knockout of multiple targets.

In the cell viability assay, nine natural products—scutellarein, emodin, bruceine D, resveratrol, hydroxysafflor yellow A, salvianolic acid B, salvianic acid A sodium, ferulic acid and glycyrrhizic acid—were experimentally assessed for their effect on the 2D cell viability of MCF-7 and Hs 578T cells. Among them, emodin, bruceine D, and scutellarein dose-dependently decreased the 2D cell viability in both cell lines. Although similar assays have previously been performed in ER+ MCF-7 cells [41], this cell line was nevertheless chosen for validation in order to be coherent with the approach taken to build the natural product models, which was based on MCF-7 cells. On the other hand, the Hs 578T cell line was chosen because of its triple-negative status and high invasiveness. To the best of our knowledge, the three drugs had not been tested before in Hs 578T, but they had been tested in other triple-negative cell lines such as MDA-MB-231, MDA-MB-453 or MDA-MB-468 [41,42,43,44,45]. Altogether, our results are in good agreement with the previously published studies. In general, we obtained higher DSS_3_ values and lower IC_50_ values in Hs 578T than in MCF-7 cells (Supplementary File S2, Table S9), indicating a higher potency for the three candidates in the triple-negative cell line compared to the ER+ cell line. In particular, bruceine D showed a submicromolar IC_50_ value in Hs 578T cells (0.71 ± 0.05 µM), suggesting that this compound might be a valuable drug for triple-negative breast cancer treatment.

Six of the nine predicted drugs did not show clear effects at the tested concentrations, and their IC_50_ values could not be determined. Resveratrol treatment decreased the Hs 578T cell viability only at a concentration of 160 μM, whereas it showed no effect on MCF-7 cells. This low effect was somewhat surprising, as previous studies have shown a decrease of MCF-7 or MDA-MB-231 survival or proliferation upon resveratrol treatment [46,47,48]. If any effect could be observed for the other five drugs, it would be a stimulatory rather than an inhibitory effect on cell viability, except for salvianolic acid B and salvianic acid A sodium, both of which decreased MCF-7 viability by ~35% at a concentration of 160 μM. Controversial results have been reported regarding the effects of these natural products on breast cancer cells. Whereas glycyrrhizic acid has been shown to induce apoptosis via ROS activation in MDA-MB-231 breast cancer cells [49], both stimulatory [50] and inhibitory effects [51] have been described for MCF-7 cell proliferation. Similarly, contradictory results have been reported regarding ferulic acid. Indeed, ferulic acid was shown to have anti-proliferative and pro-apoptotic activities in MCF-7 cells [52], and to reduce proliferation and reverse EMT in MDA-MB-231 cells [53]. In contrast, in another study, it stimulated the proliferation of MCF-7, MDA-MB-231 and other breast cancer cell lines, with the concomitant upregulation of ERα and HER2 in MCF-7 cells [54]. Hydroxysafflor yellow A has been shown to reduce the proliferation of MCF-7 cells [55]. Finally, Salvianolic acid B showed a similar inhibition of MCF-7 and MDA-MB-231 cell proliferation with a decrease in cyclin B1 levels [56]. Evidence for the anti-tumour activity of salvianolic acid B was also provided by an in vivo study, in which the drug decreased the tumour volume and increased the median survival in mice, possibly by enhancing apoptosis [57]. The observed differences between our experimental results and the literature data could be due to different experimental settings, and in a subset of the analyses, they could be due to the differential concentrations used for the treatment.

Emodin is an anthraquinone isolated from different Chinese herbs [58], and is commonly accepted as a protein tyrosine kinase inhibitor [59]. Emodin has been shown to exhibit anti-proliferation effects in several cancer types, such as breast, endometrial [60], and renal cancer [61], as well as hepatocarcinoma [62].

Scutellarein is a flavone extracted from the herb *Scutellaria baicalensis,* which induces mitochondria-mediated intrinsic apoptosis selectively on multiple myeloma [63], and inhibits the cell proliferation and EMT of hepatocellular carcinoma. Furthermore, scutellarein has been shown to inhibit the proliferation of MCF-7 and the triple-negative breast cancer cell MDA-MB-468, whereas it had no notable effect on the viability of healthy MCF-10A cells [42]. Bruceine D is a quassinoid compound extracted from the seeds of *Brucea javanica*. Bruceine D was reported to induce autophagy and apoptosis via the generation of ROS in lung cancer cells [64] and pancreatic cancer cells [65]. A bruceine D-dependent decrease in cell viability was also found in the breast cancer cell lines MCF-7 and MDA-MB-231, whereas the proliferation of healthy mammary epithelial cell MCF-10A was not affected [41]. In addition to the cell viability decrease, bruceine D was further shown to reduce the migration and invasion of MDA-MB-231 cells by upregulating the expression of E-cadherin and downregulating vimentin, suggesting a bruceine D-dependent reversal of EMT in these cells [43]. More recently, evidence was indeed provided that bruceine D treatment led to an upregulation of genes that are negatively correlated with EMT, and to a downregulation of genes that are positively correlated with EMT [66].

Taken together, the targets of emodin, scutellarein, and bruceine D, as well as their exact mode of action, are far from being completely elucidated. However, it is nowadays commonly accepted that these three drugs display anti-proliferative and anti-apoptotic effects across different cancer types, and that they counteract EMT. They induce the accumulation of ROS and regulate several signalling pathways, including PI3K/Akt/NF-κB pathways [67,68]. PI3K/AKT contributes to the regulation of survival, growth, proliferation, and metabolism [69], whereas NF-κB is implicated in cell proliferation, apoptosis suppression, angiogenesis, EMT induction, and metabolism regulation [70]. Therefore, although emodin, scutellarein, and bruceine D might not directly target metabolism, they interfere with pathways that regulate metabolism, and pathways that are related to ROS. ROS production is a double-edged sword in cancer. Indeed, depending on the concentrations and context, ROS can either promote tumour initiation and progression or inhibit tumour cell proliferation, induce apoptosis, and prevent multidrug resistance [71,72]. Cancer cells have developed strategies—such as the antioxidant system, the DNA damage repair pathway, and metabolism reprogramming—to adapt to and alleviate ROS-induced damage. However, if the ROS levels exceed a threshold level, they will overwhelm these strategies and induce oxidative stress, eventually leading to cancer cell death. Many anticancer drugs that increase ROS levels—such as 5-fluorouracil, doxorubicin, rapamycin, and erlotinib—are used in clinics [72].

In order to obtain a deeper insight into the mode of action of bruceine D, scutellarein, and emodin, we had a closer look at their results in the four in silico experiments. Among the pathways that were differentially present in the bruceine D, scutellarein and emodin models were the androgen and estrogen synthesis and metabolism pathway, as well as pathways that are required for the synthesis of estrogens, such as cholesterol metabolism, squalene and cholesterol synthesis, and steroid metabolism. Notably, the ROS detoxification pathways (in the bruceine D model) and the pathways responsible for the production of antioxidants—such as folate metabolism (emodin and scutellarein models) and heme degradation (all three models)—were downregulated, which is consistent with the described accumulation of ROS induced by the three natural products [64,65,73,74]. In order to alleviate an increase in ROS due to the lack of antioxidants, pathways that control oxidative stress—such as vitamins, NADH metabolism, and phenylalanine metabolism—were moreover predicted to be upregulated. Taken together, these three drugs show three predicted main metabolic modes of action: the downregulation of the androgen and estrogen pathway, the accumulation of ROS to initiate programmed cell death, and, to some extent, the induction of the biosynthesis pathways required for proliferation.

Increased endogenous levels of androgen and estrogen are associated with a higher risk of prostate and breast cancer development, respectively [75]. Estradiol has been shown to induce the proliferation of breast cancer cells, activate anti-apoptotic pathways, and reduce oxidative stress. Hydroxylated estrogen can further cause mutation in the DNA and induce tumours [75]. However, the effects of estrogen and estradiol vary in the function of the type of ERs at the surface of breast cancer cells. Whereas ERα acts as a tumour accelerator, Estrogen Receptor β (ERβ)—which binds to ERα, and by doing so represses ERα—is generally considered to be a tumour inhibitor. However, it has to be noted that ERβ, which is expressed in all breast cancer molecular subtypes and in the majority of breast cancer stem cells, was recently proposed as a novel therapeutic target to specifically hit stem cells [76].

Androgens have a dual and controversial effect on breast cancer development. At physiological concentrations, androgens can suppress breast cancer cell proliferation. However, at higher concentrations, androgens can promote breast cancer cell proliferation by augmenting free estrogen levels, or by regulating the transcription of genes, such as the mitotic gene Ki-67 [75].

The androgen and estrogen synthesis and metabolism pathway is targeted by 24 out of the 26 breast cancer drugs. These drugs target four reactions of this pathway that are present in the DMSO model and absent in bruceine D, emodin, and scutellarein models. Notably, one of the reactions that causes the oxidation of estrone into 16α-hydroxy-estrone in the reticulum by a monooxygenase is under the control of 13 target genes, and notably of the gene CYP3A4. CYP3A4 is targeted by 21 out of 26 cancer drugs. Hydroxylated forms of estrogen, estradiol, and testosterone were described to be genotoxic, and hence to initiate tumour formation by inducing mutations. In addition, 16α-hydroxy-estrone has a strong affinity for ERs, and the ratio between 2-hydroxy-estrone and 16α-hydroxy-estrone in the urine has, for a long time, been regarded as a biomarker for breast cancer risk, although this assumption is currently under debate [77]. Taken together, breast cancer drugs commonly target CYP3A4 and other genes of the androgen and estrogen synthesis and metabolism pathway. Furthermore, emodin and scutellarein have known targets in this pathway, including CYP3A4 in the case of emodin. CYP3A4 expression has been shown to correlate with poor overall survival in breast cancer [78,79]. However, CYP3A4 might not only induce breast cancer via hydroxylated forms of estrogen but also by the production of epoxyeicosatrienoic acids through the arachidonic metabolism pathway [79]. Both pathways are under the control of mostly the same CYP proteins that are also targets of the cancer drugs. However, the action of most of the natural products did not strongly affect the arachidonic metabolism (reactions present/absent in the DMSO were also present/absent in the natural product model). Furthermore, no difference between bruceine D, emodin, scutellarein, and the DMSO was observed for the reactions involved in the production of epoxyeicosatrienoic acids from arachidonic acid. Finally, the high number of reactions in the steroid and cholesterol pathways present in the natural product models and absent in the DMSO model, suggests that the production of estrogen might be downregulated by a depletion in steroid and cholesterol, rather than through the action of one single enzyme alone.

Taken together, bruceine D, emodin, and scutellarein are predicted to reduce the growth of MCF-7 cells through the activation of ROS with the induction of oxidative stress, and through the downregulation of the androgen and estrogen synthesis and metabolism pathway.

## 4. Methods

### 4.1. Data Retrieval

The microarray data published by Lv et al. (GEO ID: GSE85871) [18] was retrieved from Gene Expression Omnibus (GEO, http://www.ncbi.nlm.nih.gov/geo/ accessed date 20 September 2020) [80]. The dataset is a large data collection of a breast cancer cell line (MCF-7) treated with natural products. The 103 perturbations consist of 212 replicates (100 duplicates of single natural products and a duplicate of a mixture of four natural products, i.e., four replicates of glycyrrhizic acid) and six control replicates incubated with only the solvent of the drugs, i.e., DMSO (Table 3).

### 4.2. Building of Context-Specific Metabolic Models via FASTCORMICS

Gene expression CEL files were read into *RStudio* (R version 4.0.3) using the *ArrayExpress package* (version 1.50.0) (https://bioconductor.org/packages/release/bioc/html/ArrayExpress.html accessed date 3 May 2021) and the *ReadAffy* function from the *Bioconductor* repository. First, the data were log2-transformed and then normalized using the *Frozen Robust Multiarray Analysis (fRMA) package* (version 1.42.0) [81]. The barcode function from the *fRMA* package (version 1.42.0) was further run in order to compare the expression values of the dataset to a vector containing the median and standard variation of the lowest mode for each probe set for a collection of arrays for the same platform, and to compute the z-scores. The probe identifiers were converted to Entrez ID using an in-house pipeline described by [82]. In order to avoid ambiguities, probe IDs matching more than one Entrez ID were removed. The FASTCORMICS algorithm [17] (https://github.com/sysbiolux/rFASTCORMICS accessed date 3 May 2021) was used to build the context-specific models using the microarray gene expression data with the human metabolic model Recon3D [24] as inputs. In the Recon3D model, the transcript information is encoded in the model gene identifiers by the addition—i.e., .1, .2, .3—after the Entrez identifiers. As the transcript information cannot unambiguously be matched to the Ensembl identifiers, the suffixes were removed. As Recon3D is already consistent, FASTCC [83] was not run. However, for most of the reconstructions, a consistent model first has to be extracted by removing the blocked reactions identified by FASTCC before running FASTCORMICS. FASTCORMICS optionally allows us to constrain the models using medium information, and to force the inclusion of the biomass reaction or any other reactions required for modelling purposes. As the natural product-treated MCF-7 cell line was cultured in MEM/EBSS medium [18], only the input reactions for metabolites present in this media were allowed (see Supplementary File S1, Table S7 for the medium composition). The medium components were retrieved from the Cytiva website (https://www.cytivalifesciences.com/en/us/shop/cell-culture-and-fermentation/media-and-feeds/classical-media/hyclone-minimal-essential-medium-mem-variations-liquid-p-05698# related-documents accessed date 1 May 2021), and were converted into the matching metabolites names via Metabolic Atlas (version 1.7) (https://metabolicatlas.org/ accessed date 1 May 2021) [84]. Additionally, the inclusion of the biomass function in the models was forced by its addition to optional_settings.func.

### 4.3. Drug Deletion Prediction

#### 4.3.1. Natural Products

In order to predict whether knocking out the natural product targets reduces cell proliferation, the context-specific models were constrained by setting the bounds of the natural product target reactions to zero using a modified version of the *deleteModelGenes* function of the COBRA Toolbox, which can be downloaded from https://github.com/sysbiolux/Herbal_Drug_Repurpusing/blob/main/data/constraining_models/DrugDeletionCombination.m accessed date 20 March 2021. In order to obtain a list of targets for each drug, drug–target interactions information was mined from three databases: DrugBank V5 [19] (version 5.1.8), PROMISCUOUS 2.0 [20], and NPASS [21] (1.0 version). The three databases were downloaded on 12 March 2021. As UniProt IDs are used across the three databases for the target genes, non-human targets were excluded by checking the related Taxon ID (the UniProt database version in *bioDBnet* was the number 202004, released on 10 September 2020 and updated on 7 November 2020). The UniProt IDs were then converted to Entrez IDs using the *db2db* tool (*bioDBnet:* https://biodbnet-abcc.ncifcrf.gov/db/db2db.php accessed date 20 March 2021 *)*, from the biological DataBase network [85] to match the gene IDs in Recon3D. Finally, drugs with metabolic targets that are present in Recon3D were selected. The *deleteModelGenes* function of the COBRA Toolbox V3 [86] was used to set the bounds of the target reactions of natural products of the DMSO model close to zero (most of the growth rate values were around 1 or 0, with no values in between). Drugs with a grRatio of zero (no growth after drug treatment) were retained for further analysis.

#### 4.3.2. Breast Cancer Drugs

The drug deletion was repeated with the breast cancer drugs. Therefore, a list of 41 drugs approved for the treatment of breast cancer was retrieved from the NIH (https://www.cancer.gov/about-cancer/treatment/drugs/breast) on 16 March 2021 (Supplementary File S1, Table S4). Drug-target information for the breast cancer drugs was retrieved from the three databases for the drugs, and was mapped to Recon3D. As some cancer drugs were only found on the three databases as conjugates (megestrol acetate and sacituzumab govitecan), rather than the parent compound only (megestrol and sacituzumab), conjugates of the 41 cancer drugs were also retrieved from the three target databases (Table S5). In total, 26 cancer drugs could be mapped to the genes included in Recon3D. The drug deletion for the cancer drug was performed as described above, with the bounds of the breast cancer drugs’ target reactions being set to zero in the DMSO model. The DMSO model with constraints with the cancer drug targets is referred to as the cancer drug model.

#### 4.3.3. Combination of the Natural Product and Breast Cancer Drugs

Two assays were used to assess the potential synergistic effects. First, the control DMSO model was constrained (as mentioned in the Methods—Natural Products subsection) by setting the targets of each natural product and cancer drug couple to zero. The growths obtained for the drug combinations and the natural product and cancer drug alone were compared, which was performed using the *optimizeCbModel* function of the COBRA toolbox [86]. As some natural product targets might be unknown, the natural product model was constrained with breast cancer drugs as described in Methods—Breast Cancer Drugs.

#### 4.3.4. Single-Gene Deletion

An in silico single-gene deletion was performed via the *singleGeneDeletion* function from the COBRA Toolbox in order to determine whether the knockout of one of the target genes could explain the biomass reduction, or if the knockout of multiple genes was required to see an effect on growth.

### 4.4. Dissimilarity of the Natural Product Models to the DMSO Model

In order to assess the impact of the natural products on the metabolism of breast cancer cells, a dissimilarity score D was computed based on the Jaccard Similarity Index J (D = 1-J). The dissimilarity score is equal to 1 if the number of shared reactions between two models is above the total number of reactions. Using the *dist* function inside the *proxy package* (version 4.0.3) in *Rstudio* (version 4.0.3), the Jaccard index was calculated between every natural product and the DMSO model, and then converted into the dissimilarity score. The natural products were then sorted by their dissimilarity to the DMSO model reactions and the top-five natural products were selected, as there was a larger drop of dissimilarity between the fifth and sixth natural product models.

### 4.5. Similarity between the Natural Product and Breast Cancer Drug Model Fluxes

In order to identify the natural products that have a similar effect on the MCF-7 metabolism as the breast cancer drugs, a Flux Variability Analysis (FVA) [87] was performed using the *fluxVariability* function in the COBRA toolbox.

A dissimilarity score was computed between the flux range of the cancer drug models and the DMSO model (dissimilarity score = 1 − si, where si is equal to $si=mean(\frac{max\left(0,\left(min\left(v1max,v2max\right)-max\left(v1min,v2min\right)+epsilon\right)\right)}{\left(max\left(v1max,v2max\right)-min\left(v1min,v2min\right)+epsilon\right))}$***)***.

If the reactions in both models have very similar flux ranges (the difference being smaller than epsilon, which is usually set to 1 × 10^−4^), then the dissimilarity score is equal to 0. If the reactions shared by both have very different ranges, the dissimilarity score will tend to 1. In case a reaction was not included in one of the two compared models, the bounds were considered to be equal to zero. Based on their dissimilarity scores, two breast cancer drugs were selected: capecitabine and methotrexate (see Figure 2A for cancer drugs with dissimilarity scores > 1 × 10^−4^). Then, in a second step, the similarity score (*si)* was computed between the natural product and the capecitabine and methotrexate models (Figure 2B).

### 4.6. Pathway Analysis

In order to identify the pathways that are more preferably targeted by breast cancer drugs, the drug targets were identified as described in Methods—Cancer Drugs. These target genes were mapped to the gene identifiers of Recon3D, and the target reactions were obtained using the *rxnGeneMat* field of Recon3D. These target reactions were then assigned to a pathway using the *subSystem* field of Recon3D. The number of cancer drugs targeting a pathway was plotted against the number of natural products. In order to take into account the size of the pathways, the number of drugs was normalised based on the number of reactions in the pathway under gene control, and these rates were represented in the colour code of the plot. Eight pathways that are targeted by the most approved breast cancer drugs were selected for their potential anticancer action. Among the eight drugs, the drugs that showed the greatest impact on the metabolism of breast cancer were selected by computing the difference of the rate of the reaction presence between the natural product and the cancer models (the rate of the reactions in a pathway that are present in a natural product model and absent in the DMSO model, and vice versa). First, the rate of reactions present per pathway was calculated for each model and each pathway by dividing the number of active reactions in the context-specific models by the total number of reactions in this pathway in Recon3D using the subSystems field. Then, the difference rate of reaction presence was computed between each natural product model and the DMSO model. The pathways were then sorted in the function of the interquartile range. All of the pathways with three reactions or fewer were excluded.

### 4.7. Drug Selection

In total, 23 drugs were predicted by at least one of the four previous steps (drug deletion, the dissimilarity to DMSO, the similarity to breast cancer drugs, and the pathway analysis). The drugs predicted by two or more experiments were in general selected, unless they were toxic or had already been tested in MCF-7 cells (Table 2, green). In order to refine the list of drugs for the experimental validation, the drugs that passed one test but which had some pre-clinical and literature information supporting a potential use in cancer, in general, were also selected. In order to assess whether the drug candidates were already tested for breast cancer or any other cancer type, the Dr. Duke Database [30] (downloaded on 4 November 2020) and the Drug Repurposing Hub [31] (downloaded on 24 May 2021) were downloaded, and clinicalTrials.gov (https://clinicaltrials.gov accessed date 1 June 2021) [88] was mined (see Supplementary File S1, Table S8). The 102 Chinese natural products were mapped in the Dr. Duke database. In total, 23 natural products had a phytochemical classification inside the database, and 44 natural products were mapped to 358 unique activities. The Drug Repurposing Hub repository was used to find information, e.g., the mode of action for natural products, and the clinical trials’ phases were mined to find potential closed or ongoing trials. The preclinical in vitro testing data for the 23 drugs were retrieved from different sources; the compiled data, as well as the link to the data source, are found in Supplementary File S1, Table S6. Based on this data mining, two drugs were removed from the list due to high toxicity (strychnine) and due to the abundance of existing experimental treatments on breast cancer cell lines (narciclasine) (Table 2, red). However, due to the evidence retrieved from the data mining and literature search, four drugs (bruceine D, emodin, salvianic acid A sodium, and scutellarein) that were predicted by only one step were added to the final list of drugs (Table 2, blue). Resveratrol, similar to narciclasine, had already been tested before in more than three breast cancer cell lines, and it is considered to be one of the most promising anticancer natural products. Only resveratrol was kept in the final list of drugs as a positive control, while narciclasine was excluded.

### 4.8. Experimental Validation

#### 4.8.1. Natural Products and Inhibitors

Salvianic acid A sodium (# SML0679, Merck KGaA, Darmstadt, Germany) was prepared in growth medium and ferulic acid (# HY-N0060), glycyrrhizic acid (# HY-N0184), scutellarein (# HY-N0752), hydroxysafflor yellow A (# HY-N0567), salvianolic acid B (# HY-N1362), emodin (# HY-14393), bruceine D (# HY-N3014) and resveratrol (# HY-16561) (all from MedChemExpress, Monmouth Junction, NJ, USA) were prepared in DMSO at an initial concentration of 100 mM, and then stored in aliquots at −80 °C. A 10 mM stock solution of ophiobolin A (# sc-202266, Santa Cruz Biotechnology, Inc., Heidelberg, Germany) was prepared in DMSO, and then stored in aliquots at −20 °C. A 100 mM stock solution of benzethonium chloride (# 53751, Merck) was prepared in H_2_O, and then stored in aliquots at −20 °C.

#### 4.8.2. Cell Culture

The MCF-7 (# HTB-22) and Hs 578T (# ACC 781) cells were obtained from American Type Culture Collection (Manassas, VA, USA) and from Leibniz Institute DSMZ-German Collection of Microorganisms and Cell Cultures GmbH (Braunschweig, Germany), respectively. Both cell lines were cultured in Dulbecco’s Modified Eagle’s Medium (DMEM) (Lonza Group, Basel, Switzerland) supplemented with 10% fetal bovine serum and 2 mM L-glutamine (complete DMEM) (Lonza Group). The cells were routinely passaged and grown at 37 °C in a 5% CO_2_, H_2_O-saturated atmosphere.

#### 4.8.3. 2D Cell Viability Assay (2D Cell Proliferation)

The 2D cell viability was assessed using the alamarBlue^TM^ cell viability reagent (# DAL1100, Thermo Fisher Scientific, Waltham, MA, USA) according to the manufacturer’s protocol. Briefly, MCF-7 or Hs 578T cells were seeded at a density of 3000 cells in 100 μL complete DMEM medium per well in 96-well flat-bottom culture plates.

After a 24-h incubation at 37 °C, the cells were treated with DMSO as a vehicle control, with 200 μM benzethonium chloride (BzCl) as a positive control for cell death, or with natural test products diluted in complete DMEM. The treatment with natural products was performed in a 1:2 dilution series ranging from 160 μM to 0.625 μM, or from 20 μM to 0.078 μM for bruceine D. Ophiobolin A, used here as a control compound for its anti-proliferative activities, was used for treatment in a 1:2 dilution series ranging from 20 μM to 0.078 μM.

After a 72 h treatment at 37 °C, alamarBlue^TM^ cell viability reagent was added to each well to a final concentration of 10%, and the plate reading was performed after a 2.5-h incubation in the dark at 37 °C. The fluorescence intensity (excitation at 530 ± 15 nm and emission at 590 ± 20 nm) was measured using a CLARIOstar (BMG LABTECH, Ortenberg, Germany) plate reader.

#### 4.8.4. Drug Sensitivity Score Determination and Data Analysis

The raw fluorescence intensity values obtained from the cell viability assays were analysed using the DSS pipeline website BREEZE (https://breeze.fimm.fi accessed date 14 December 2021) [34,89]. The absolute IC_50_ and DSS_3_ values were derived from the data analysis, with DSS being a more robust parameter to quantitate drug sensitivity than IC_50_, corresponding essentially to the normalized area under the curve (AUC) of dose–response data [35].

Prism 9.2.0 (GraphPad Software, San Diego, CA, USA) was used to establish the dose–response curves. The results are presented as the mean ± SEM of at least three independent biological replicates, unless otherwise indicated. Each biological replicate was carried out in three technical replicates, out of which at least two were considered for evaluation.

### 4.9. Post-Experimental Analysis of the Pathways Affected by Bruceine D, Scutellarein, and Emodin

In order to gain insight into the possible mode of action of bruceine D, scutellarein and emodin, pathway analyses were performed on the three natural product models, which were then compared against the DMSO model, at a pathway level and a reaction level. Furthermore, targeted reactions by emodin and scutellarein were compared with reactions targeted with cancer drugs. First, the differences in the reaction presence rate between the three drugs and the DMSO model were computed. The pathways with absolute differences below 0.1 were not plotted in Figure 6 but are shown in Supplementary File S2, Figure S6. Second, the target genes of the 24 cancer drugs, emodin, and scutellarein, were plotted against the eight most commonly targeted pathways of breast cancer drugs (androgen and estrogen synthesis and metabolism, arachidonic acid metabolism, bile acid synthesis, cytochrome metabolism, drug metabolism, linoleate metabolism, steroid metabolism, transport, and extracellular pathways) (see Figure 7 and Supplementary File S2, Figure S7). The size of the dots represents the number of drugs targeting the genes, and the numbers correspond to the number of reactions in the pathways under the control of the target gene. Bruceine D was excluded from this analysis because of the lack of target information. Third, the drug targets were plotted against the reactions that were differentially present between each natural product model and the DMSO model for each of the eight most commonly targeted pathways. The reactions present in the natural product models and absent in DMSO are depicted in red, and vice versa in blue (see Supplementary File S2, Figure S8).

## 5. Conclusions

In conclusion, our in silico analysis pipeline (https://github.com/sysbiolux/Herbal_drug_prediction, accessed on 30 December 2021) enabled us to narrow down a list of 101 single natural products and one mixture of natural products to nine anticancer drug candidates. The subsequent experimental validation allowed us to confirm the potency of three out of these nine candidates, namely emodin, scutellarein, and bruceine D. Finally, our approach led to the identification of the pathways which are potentially involved in the anticancer activity of the three candidates.

## Figures and Tables

**Table 1:** Summary of the 103 context-specific models (101 natural products, one mixture of natural products, and one DMSO model) in terms of the median number of reactions, metabolites, and genes.

	Reactions	Metabolites	Genes
Median	1895 ± 87	1593 ± 66	1036 ± 49
Min	1593	1353	908
Max	2169	1790	1128

**Table 2:** Summary table of the 23 candidate natural products for potential anticancer action based on four in silico analysis steps. Highlighted in green are the drugs that were predicted in two steps; in blue are the drugs predicted in only one step, but that were additionally supported by data from the Dr. Duke Database and the Drug Repurposing Hub Database. Both sets were submitted for experimental validation. Highlighted in red is the drug strychnine, which was eliminated due to toxicity; highlighted in orange is the drug narciclasine, which was removed due to a large amount of published data. In yellow are the drugs that were predicted by one step and not retained for validation. The background colors correspond to the different NP selection criteria, which are mentioned in the main text as (Table 2, COLOR). These colors can be replaced with special characters (for example: †, ‡, §, ||) by added them to the 1st column (Ferulic Acid†), but then the refernce in the main text would need to change to (Table 2, SYMBOL).

	Drug Deletion	Dissimilarity to DMSO Model	Similarity to Cancer Drug Models	Pathway Analysis	Anticancer Activity (Dr. Duke Database)	Mode of Action (Drug Repurposing Hub Database)	Clinical Phase
Ferulic Acid	1	1	1			Antioxidant	Phase 2
Glycyrrhizic acid			1	1			
Resveratrol	1			1	Antitumor, Antioxidant, Apoptotic, Antiangiogenic	Cytochrome P450 inhibitor, SIRT activator	Launched
Scutellarein				1	Antioxidant, Cancer-preventive		
Strychnine				1	Antioxidant	Acetylcholine receptor antagonist	Preclinical
Narciclasine		1		1			
Hydroxysafflor yellow A		1	1			Anti-tumor agent	Preclinical
Salvianolic Acid B		1	1			EGFR inhibitor, metalloproteinase inhibitor	Phase 2
Daidzin			1		Antioxidant, Cancer-preventive	Antioxidant	Phase 1
Macrozamin			1				
Chelerythrine				1	Antimitotic		
Chenodeoxycholic acid				1		11-ß hydroxysteroid dehydrogenase inhibitor, FXR agonist	Launched
Emodin				1	Antitumor, Immunosuppressant	11-ß hydroxysteroid dehydrogenase inhibitor	Preclinical
Tetrahydropalmatine				1			
Bacopaside I			1				
Ethyl caffeate			1				
Ginsenoside Rb1			1				
Hypaconitine			1				
Salidroside			1			Beta-amyloid protein neurotoxicity inhibitor	Preclinical
Salvianic acid A sodium			1			Matrix metalloprotease inhibitor	
Schizandrin			1		Antioxidant		
Bruceine D		1				Glycine receptor antagonist	Preclinical
Osthole				1		Calcium channel blocker	Preclinical

**Table 3:** Number of replicates for each condition.

Perturbations	Number of Replicates
DMSO	6
A mixture of four natural products (tanshinone IIA, salvianic acid A sodium, protocatechuic aldehyde, salvianolic acid B)	2
101 natural products	2 (except for glycyrrhizic acid that has 4)

## Data Availability

Data is contained within the article and supplementary materials.

## Supplementary Materials

The following supporting information can be downloaded at https://www.mdpi.com/article/10.3390/ph15020179/s1. Table S1: Detailed model statistics for the 103 context-specific natural product models; Table S2: Natural product target interactions extracted from DrugBank V5, PROMISCUOUS 2.0 & NPASS; Table S4: List of approved breast cancer from cancer.gov; Table S5: Breast Cancer drug-target interactions extracted from DrugBank V5, PROMISCUOUS 2.0 & NPASS; Table S6: Preclinical *in vitro* evidence for the candidate natural products; Table S7: Medium composition of the MEM medium used for context-specific model building; Table S8: Additional clinical evidence for the candidate natural products; Table S10: Published concentrations and IC_50_ values for bruceine D, scutarellein and emodin; Table S11: List of reactions targeted by emodin, scutellarein, bruceine D and the breast cancer drugs in the androgen and estrogen metabolism and synthesis pathway; Figure S1: Dissimilarity score for the 102 natural product models; Figure S2: Similarity score between the natural product models and methotrexate (blue) and capecitabine (red); Figure S3: Chondroitin synthesis, folate metabolism, histidine metabolism, and androgen and estrogen synthesis and metabolism are the top four pathways showing the highest difference of reaction presence rate between the natural product and DMSO models (Highest rate of reaction that is present in natural product models and absent in DMSO and vice versa); Figure S4: Ten out of the 12 natural products that target the androgen and estrogen synthesis and metabolism pathway, have differences in reaction presence rate between the natural product and DMSO models; Figure S5: Resveratrol, hydroxysafflor yellow A, salvianolic acid B, salvianic acid A sodium, ferulic acid, and glycyrrhizic acid do not notably affect 2D breast cancer cell viability; Figure S7: Target genes and number of affected reactions of emodin and scutellarein; Figure S8: Natural product models reactions for bruceine D, emodin and scutellarein that are differently present between the three natural product and DMSO models and that are under the control of breast cancer drugs;Table S3: Number of natural products and cancer drugs with drug-target interaction extracted from three databases;Table S9: DSS_3_ and IC_50_ values for the control compound ophiobolin A and each natural product tested in Hs 578T or MCF-7 cells (mean ± SEM). ND = Not determined.
