# Supplementary material for: Bruceine D Identified as a Drug Candidate against Breast Cancer by a Novel Drug Selection Pipeline and Cell Viability Assay
Source: Pharmaceuticals (Basel). 2022 Jan 31;15(2):179. doi: 10.3390/ph15020179 (PMC8875459; doi:10.3390/ph15020179)
Supplement: Supplementary file 1 [file pharmaceuticals-15-00179-s001.zip › Supplementary/Supplementary File S2.pdf]

Supplementary File S2

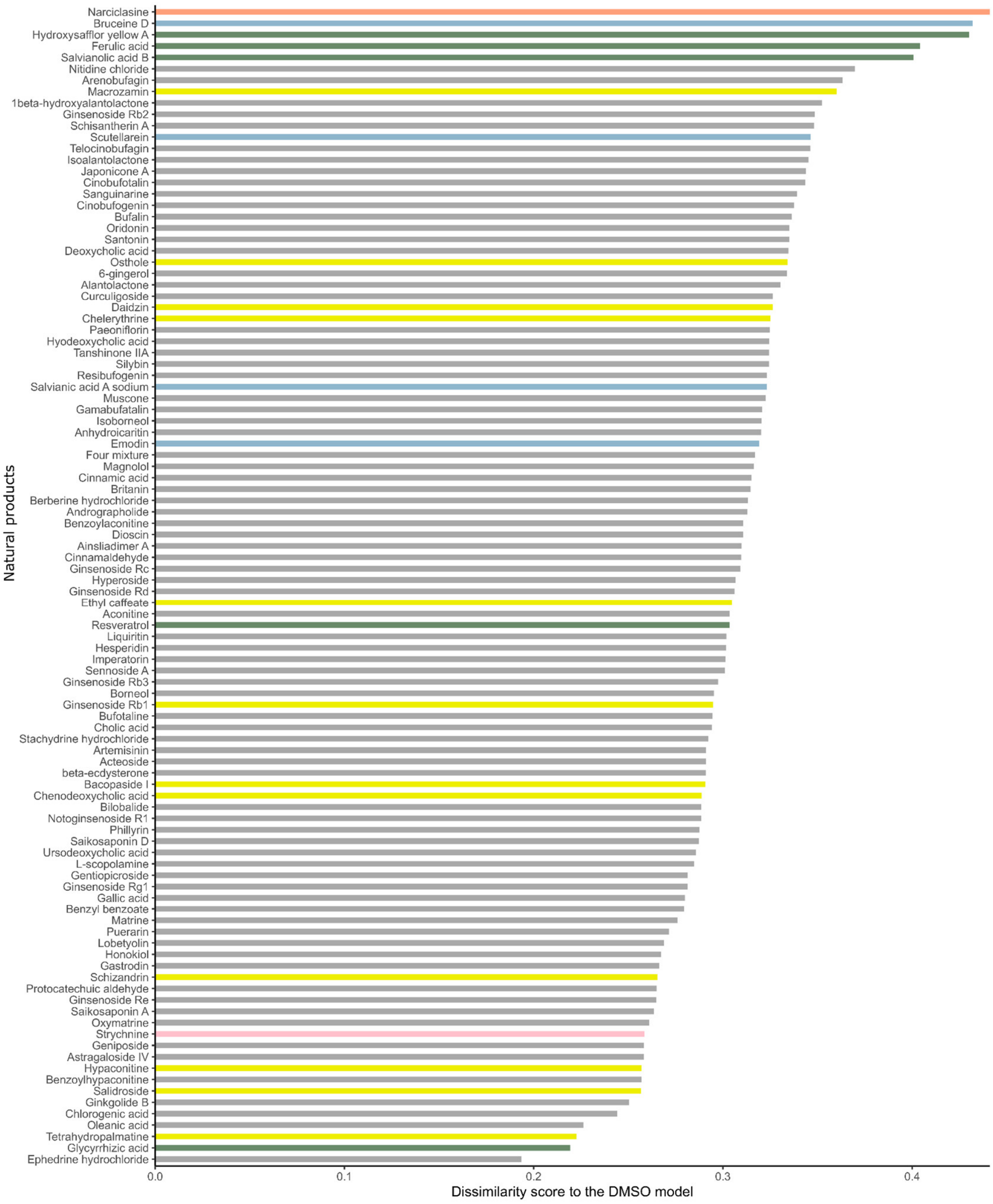

Figure S1: Dissimilarity score for the 102 natural product models

Natural products are sorted based on their dissimilarity score. Drugs coloured in green were selected for the validation as they were predicted in more than one of the *in silico* steps of the analysis workflow. The blue colour marks drugs that were predicted by one of the *in silico* steps but that were selected for validation as they were supported by evidence found in databases and literature. Yellow stands for drugs not retained for validation as only predicted by one experiment, in grey are the drugs that were not predicted by any experiments, red and orange stands for reactions that were rejected due to toxicity and multitude of published evidence, respectively.

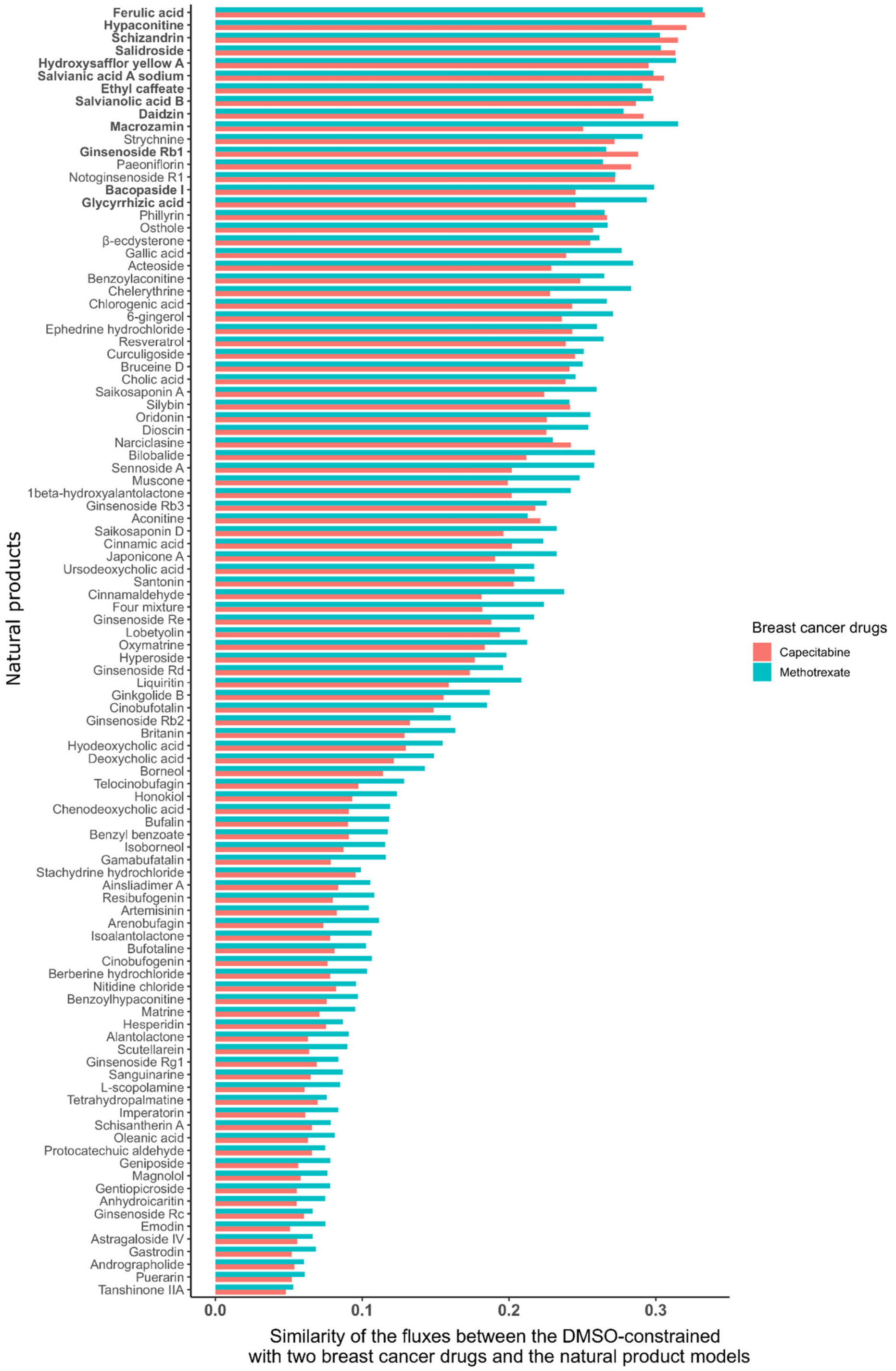

Figure S2: Similarity score between the natural product models and methotrexate (blue) and capecitabine (red). A similarity score between the flux ranges of the natural product models and the two DMSO models constrained with methotrexate and capecitabine obtained by Flux Variability Analysis (FVA) was computed. The natural products are sorted by their average similarity score of both methotrexate and capecitabine. The top ten most similar natural products to methotrexate and another set of ten drugs with the highest similarity to capecitabine were selected (13 natural products in total as some drugs were similar to both cancer drugs, **see Table 2**) as potential anticancer drugs.

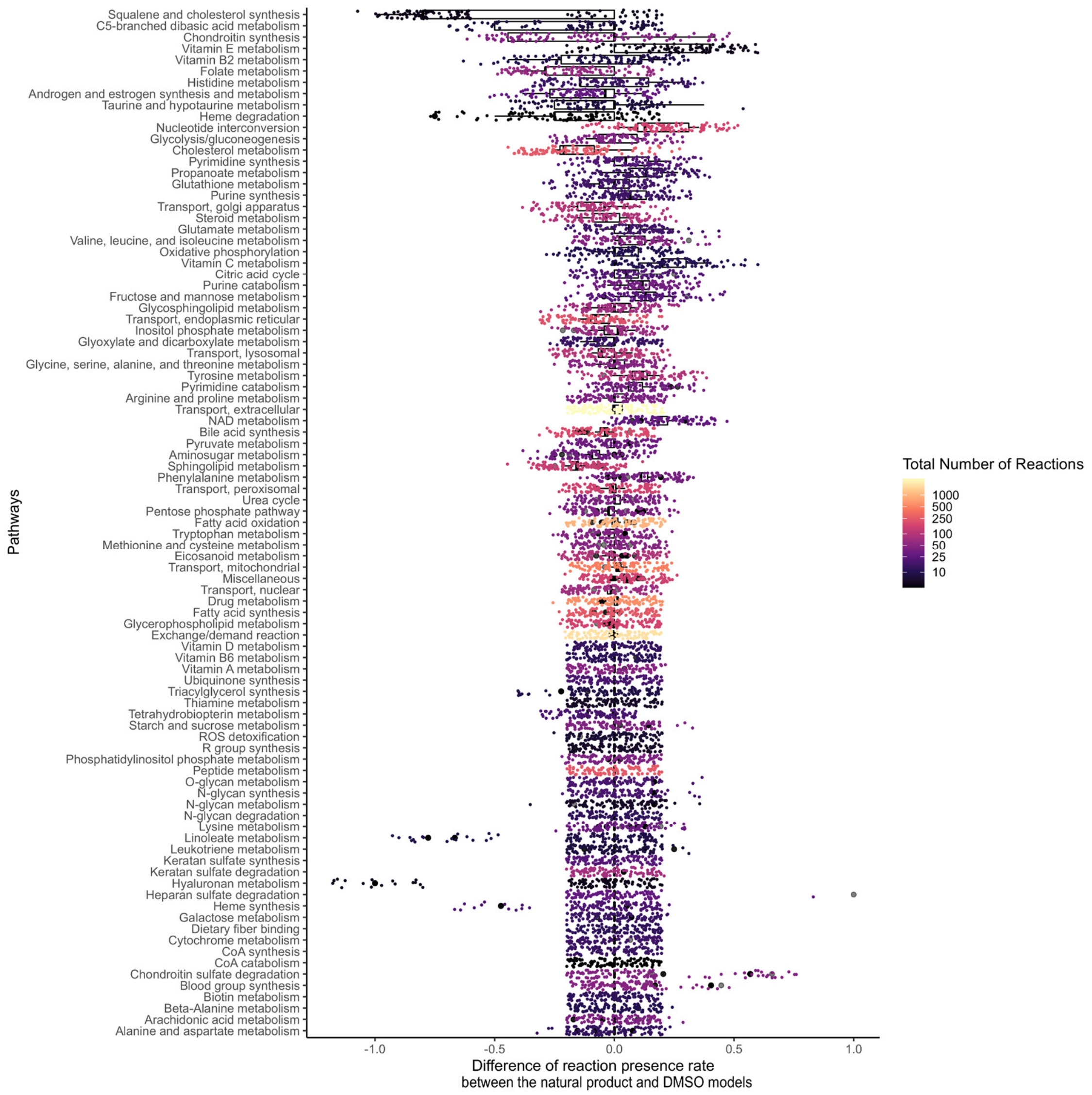

Figure S3: Chondroitin synthesis, folate metabolism, histidine metabolism, and androgen and estrogen synthesis and metabolism are the top four pathways showing the highest difference of reaction presence rate between the natural product and DMSO models (Highest rate of reaction that is present in natural product models and absent in DMSO and vice versa).

The rate of present reactions per pathway was calculated for each natural product and each pathway by dividing the number of active reactions in the context models by the total number of reactions in this pathway. Then, the difference in reaction presence rate between natural product models and the DMSO model was calculated. Pathways with three reactions or less were discarded. The pathways were colourized with a total number of reactions in each pathway. Pathways were sorted by the interquartile range of the reactions per pathway difference.

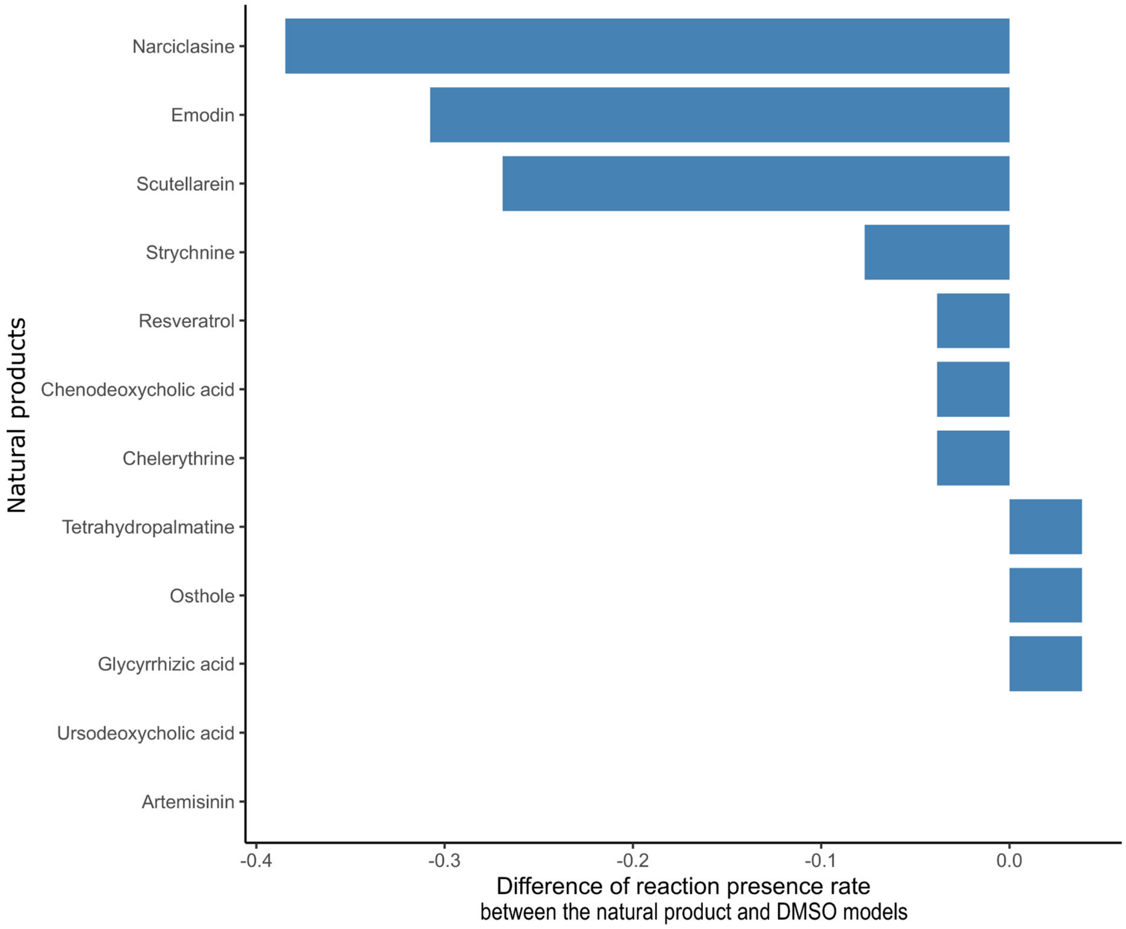

Figure S4: Ten out of the 12 natural products that target the androgen and estrogen synthesis and metabolism pathway, have differences in reaction presence rate between the natural product and DMSO models.
The androgen and estrogen synthesis and metabolism pathways were used to select natural products with potential anticancer effects (see **Figure 4**). The difference in reaction presence rate between the natural product and DMSO model was calculated as in **Figure 5**. Out of the 12 drugs targeting this pathway, only ten natural products show differences in reaction presence rate against DMSO models, while two natural products (ursodeoxycholic acid and artemisinin) show no difference, suggesting that these drugs do not affect this pathway.

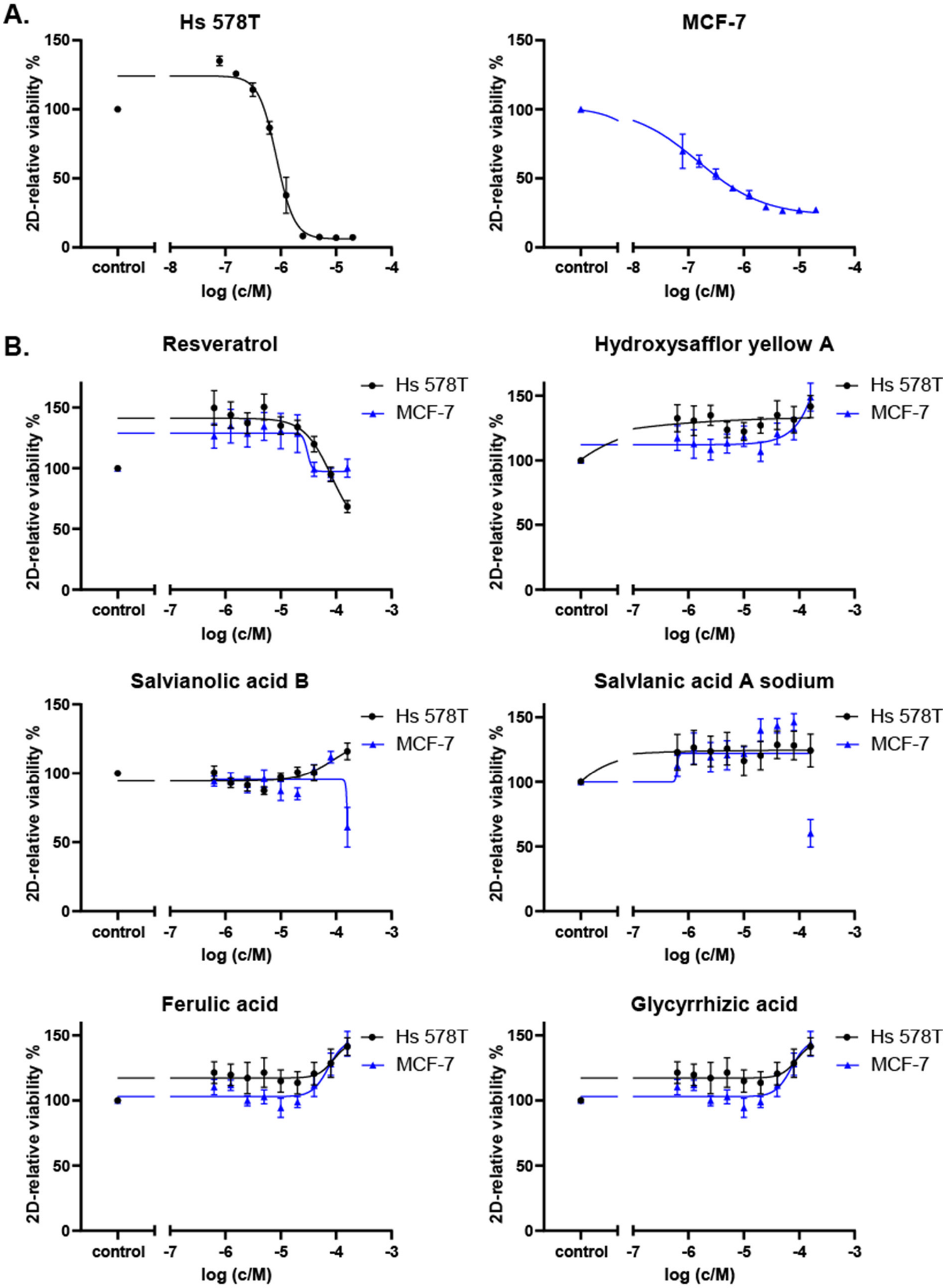

Figure S5: Resveratrol, hydroxysafflor yellow A, salvianolic acid B, salvianic acid A sodium, ferulic acid, and glycyrrhizic acid do not notably affect 2D breast cancer cell viability.

A. Dose-dependent decrease of the 2D cell viability of both MCF-7 and Hs 578T breast cancer cell lines after a 72 h treatment with the control compound ophiobolin A. Treatment was performed in a 1:2 dilution series ranging from 20 µM to 0.078 µM (n = 2). B. Among the natural products resveratrol, hydroxysafflor yellow A, salvianolic acid B, salvianic acid A sodium, ferulic acid and glycyrrhizic acid, only resveratrol induced a minor dose-dependent

decrease of 2D cell viability of Hs 578T breast cancer cells after 72 h. Treatment was performed in a 1:2 dilution series ranging from 160 µM to 0.625 µM for all the tested natural products (n = 3).

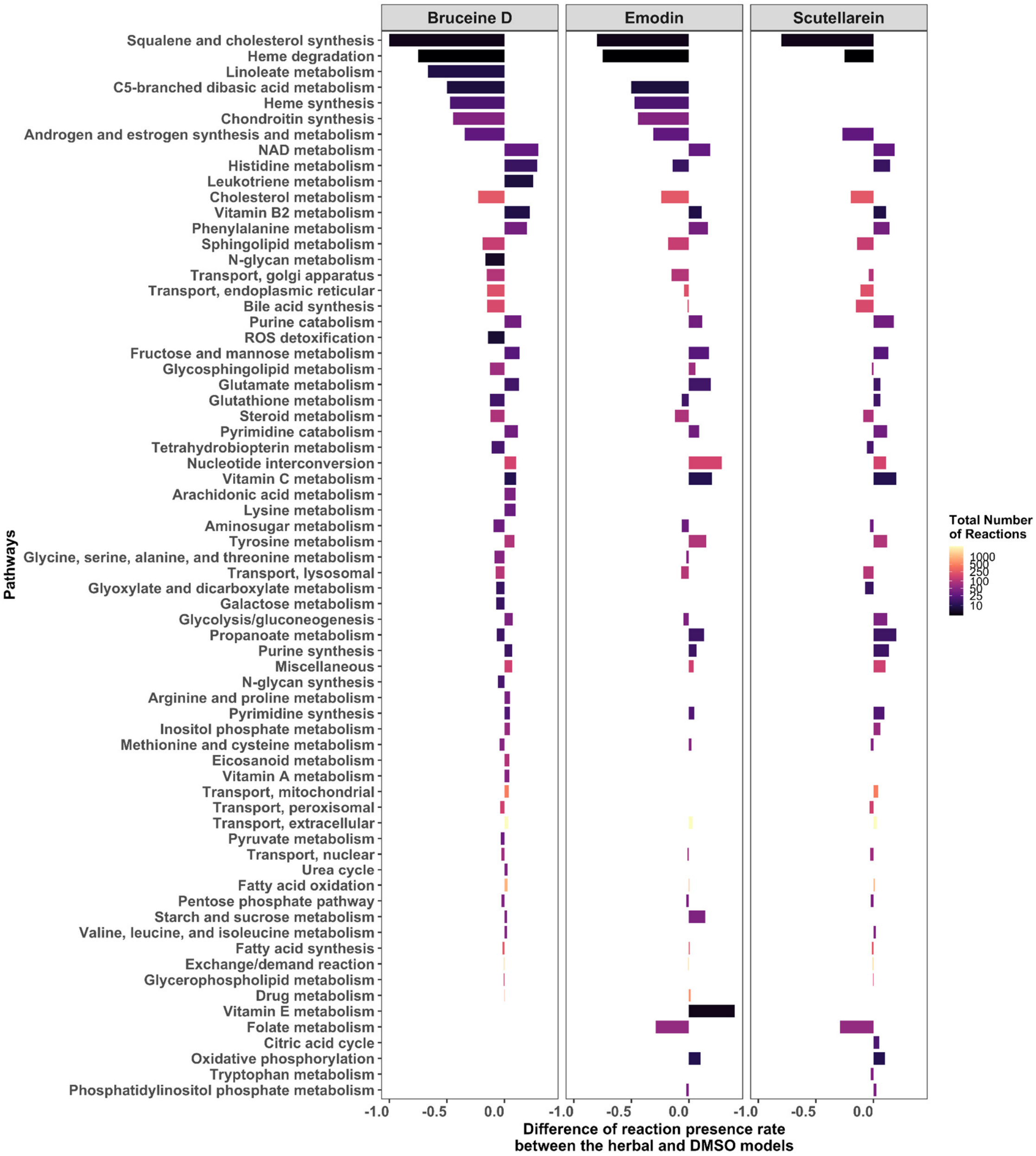

Figure S6: Difference of reaction presence rate between the three natural products **with effect on cell viability assay, compared to the DMSO model on all pathways.** Differences in reaction presence rate between the natural products (bruceine D, emodin, and scutellarein) and the DMSO model were calculated (see Figure 6). Only pathways with zero difference to the DMSO model for all the three natural product models were excluded.

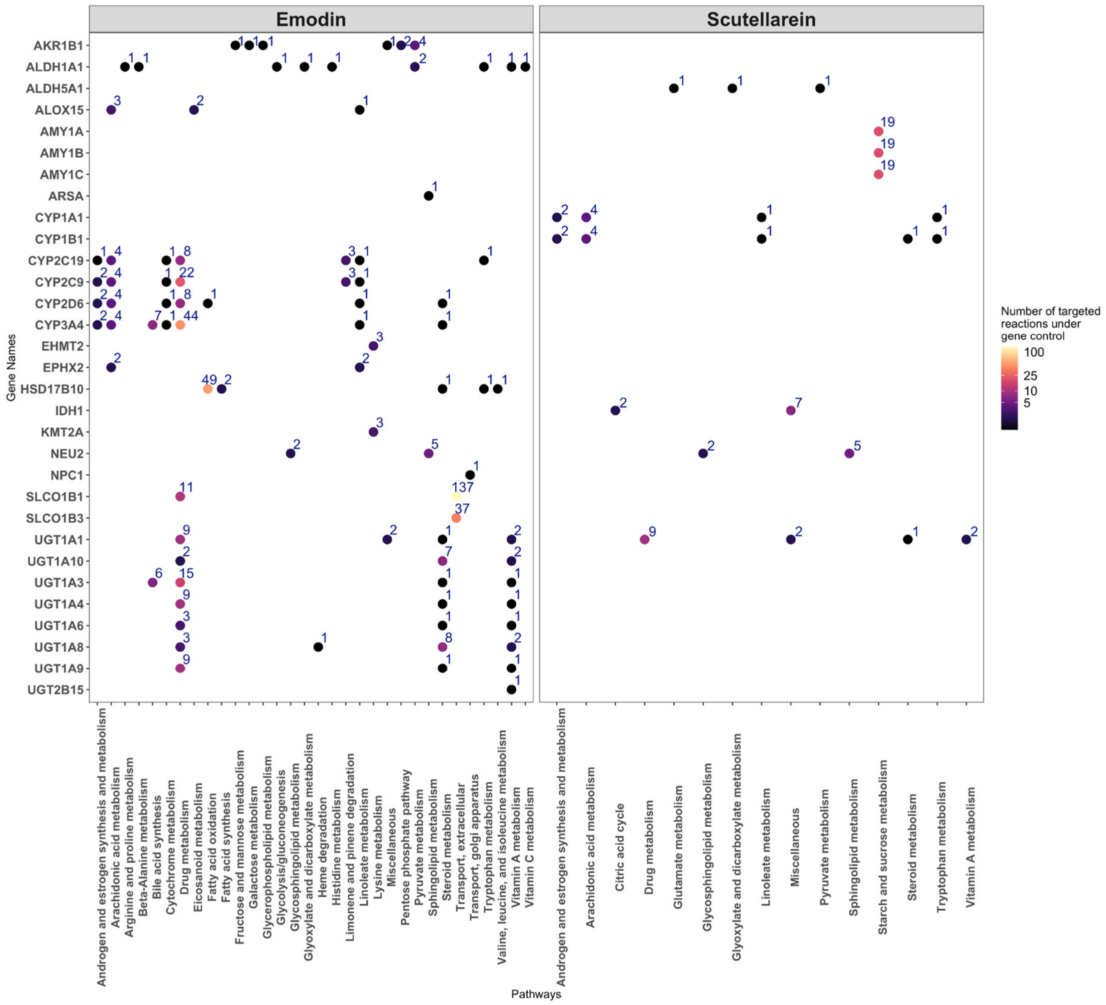

Figure S7: Target genes and number of affected reactions of emodin and scutellarein.

Target genes of emodin and scutellarein were plotted against the eight most targeted pathways. The size of the dots represents the number of breast cancer drugs that target a gene, whereas the numbers stand for the number of reactions under the control of the genes targeted by emodin and scutellarein.

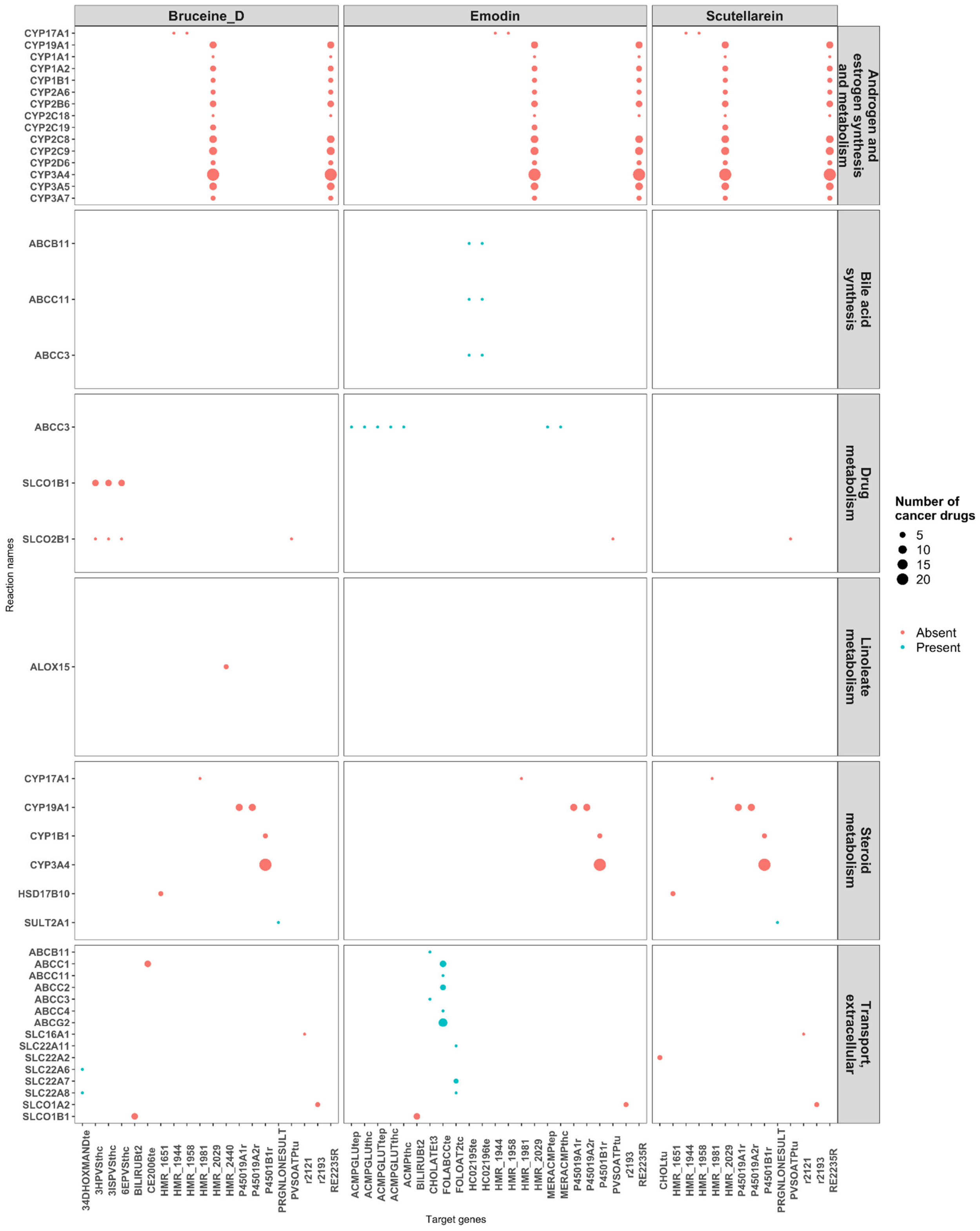

Figure S8: Natural product models reactions for bruceine D, emodin and scutellarein that are differently present between the three natural product and DMSO models and that are under the control of breast cancer drugs. Reaction presence for the models of bruceine D, emodin, scutellarein and DMSO were retrieved for the eight pathways that are targeted by most cancer drugs. One pathway (Cytochrome metabolism) was inactive in the four models. The difference in the reaction presence between the three natural products and the DMSO models were

calculated. The red dots represent reactions that are active in the DMSO model and inactive in the natural product models and vice versa for green dots. Reactions that were present or absent in all natural products and DMSO models were excluded. Thus, Chondroitin metabolism was excluded as all the active reactions in the DMSO model are also active in the natural product models. Then, the reactions of the three models were mapped with the reactions of the cancer drug targets to determine how many cancer drugs can target each reaction-gene pair.

Supplementary File S2 Table S3. Number of natural products and cancer drugs with drug-target interaction extracted from three databases.

		Cancer drugs	Natural products
The total number of drugs		41	101 single natural products + 1 mixture
Drug-target databases	DrugBank V5	31	13
	PROMISCUOUS 2.0	33	16
	NPASS	2	40
Across the three databases		36	47
After selecting human genes		36	44
After selecting metabolic targets		26	35

Supplementary File 2 Table S9. DSS_3 and IC_{50} values for the control compound ophiobolin A and each natural product tested in Hs 578T or MCF-7 cells (mean ± SEM). ND = Not determined.

Natural products	Hs 578T		MCF-7	
	DSS_3	IC_{50} (µM)	DSS_3	IC_{50} (µM)
Scutellarein	29 ± 17	47 ± 29	9 ± 4	49 ± 17
Emodin	24 ± 9	28 ± 9	6 ± 2	65 ± 12
Bruceine D	47 ± 3	0.71 ± 0.05	45 ± 8	9.5 ± 7.7
Resveratrol	1.9 ± 1.6	ND	2.7 ± 1.2	ND
Hydroxysafflor yellow A	6 ± 3	ND	0	ND
Salvianolic acid B	0	ND	0.4 ± 0.2	ND
Salvianic acid A sodium	0	ND	0.3 ± 0.2	ND
Ferulic acid	0	ND	0	ND
Glycyrrhizic acid	0	ND	0	ND
Control compound	Hs 578T		MCF-7	

	DSS_3	IC_{50} (μM)	DSS_3	IC_{50} (μM)
Ophiobolin A	35 ± 1	1.05 ± 0.06	82 ± 3	0.13 ± 0.05
